# Monocyte Chemoattractant Protein-1 stimulates the differentiation of rat stem and progenitor Leydig cells during regeneration

**DOI:** 10.1186/s12861-020-00225-1

**Published:** 2020-10-06

**Authors:** Xiangcheng Zhan, Jingwei Zhang, Saiyang Li, Xiaolu Zhang, Linchao Li, Tiantian Song, Qunlong Liu, Jun Lu, Yunfei Xu, Ren-Shan Ge

**Affiliations:** 1 Department of Urology, Shanghai Tenth People’s Hospital, Tongji University School of Medicine, Shanghai, 200072 China; 2 Tongji University School of Medicine, Shanghai, 200092 China; 3 Department of Urology, Yijishan Hospital, Wannan Medical College, Wuhu, 241000 Anhui China; 4 Nanjing Medical University, Nanjing, China; 5 Department of Anesthesiology, the Second Affiliated Hospital and Yuying Children’s Hospital of Wenzhou Medical University, Wenzhou, Zhejiang, 325027 China

**Keywords:** Monocyte chemoattractant protein-1, Stem Leydig cells, Proliferation, Differentiation, Testosterone

## Abstract

**Background:**

Monocyte chemoattractant protein-1(MCP-1) is a chemokine secreted by Leydig cells and peritubular myoid cells in the rat testis. Its role in regulating the development of Leydig cells via autocrine and paracrine is still unclear. The objective of the current study was to investigate the effects of MCP-1 on Leydig cell regeneration from stem cells in vivo and on Leydig cell development in vitro.

**Results:**

Intratesticular injection of MCP-1(10 ng/testis) into Leydig cell-depleted rat testis from post-EDS day 14 to 28 significantly increased serum testosterone and luteinizing hormone levels, up-regulated the expression of Leydig cell proteins, LHCGR, SCARB1, CYP11A1, HSD3B1, CYP17A1, and HSD17B3 without affecting progenitor Leydig cell proliferation, as well as increased ERK1/2 phosphorylation. MCP-1 (100 ng/ml) significantly increased medium testosterone levels and up-regulated LHCGR, CYP11A1, and HSD3B1 expression without affecting EdU incorporation into stem cells after in vitro culture for 7 days. RS102895, a CCR2 inhibitor, reversed MCP-1-mediated increase of testosterone level after culture in combination with MCP-1.

**Conclusion:**

MCP-1 stimulates the differentiation of stem and progenitor Leydig cells without affecting their proliferation.

## Background

Testosterone is critical for maintaining the secondary sexual characteristics and promoting spermatogenesis in adult males [1]. Approximately 95% of circulatory testosterone amount is contributed by the Leydig cells, which are located in the interstitium of the adult testis. To maintain the normal testosterone production, certain numbers of Leydig cells and the full-scale capacity of steroidogenesis per se are required by each testis. These properties of Leydig cells are achieved via their pubertal development. Although pituitary luteinizing hormone (LH) is important for maintaining the full capacity of androgen production and the late-stage development of Leydig cells after it binds its receptor (LHCGR), many other growth factors and cytokines are also involved [1]. However, the regulation of Leydig cell development by these growth factors and cytokines is not well investigated. Our research group previously isolated and identified a platelet-derived growth factor receptor α (PDGFRA)-positive stem Leydig cell, which is capable of committing into the Leydig cell lineage [2]. These stem Leydig cells are located in the interstitium of the testis and can commit into the Leydig cell lineage after the treatment of ethane dimethane sulfonate (EDS), an alkylating drug that selectively eliminates all Leydig cells in rat testis [3].

After comparing the transcriptome of cells in the Leydig cell lineage with that of PDGFRA-positive stem Leydig cells, a series of growth factors and cytokines were identified in stem Leydig cells [4]. One of these cytokines is monocyte chemoattractant protein-1 (MCP-1, also called CCL2), which is abundantly expressed in stem Leydig cells and progressively declines with the development of the Leydig cells [4]. Its receptor is the C-C chemokine receptor type 2 (CCR2), which is a typical G protein coupled receptor on the target cell membrane [5]. Testicular peritubular myoid cells also express MCP-1 and several cytokines such as interleukin-1 and tumor necrosis factor-α (TNF-α) that can dramatically upregulate expression of MCP-1 in vitro [6]. In the experimental autoimmune orchitis of rats, MCP-1 expression was increased in Leydig and peritubular, mononuclear, and endothelial cells and also detected in Sertoli cells in severe orchitis [7]. Inflammation was also related with the release of MCP-1 in rats [8]. However, whether MCP-1 has autocrine and paracrine effects on the Leydig cell development and regeneration and its potential mechanism remains unclear.

Conceptually, the regeneration of Leydig cells in the adult rat testis when all Leydig cells are eliminated by EDS is divided into four phases: stem, progenitor, immature, and adult Leydig cells [3, 9]. Seven days after one intraperitoneal injection of EDS, all Leydig cells are eliminated 7 d after EDS, with only stem Leydig cells being present in the interstitium [3]. Stem Leydig cells do not express Leydig cell biomarkers such as LHCGR and 3β-hydroxysteroid dehydrogenase 1 (HSD3B1) [3]. From post-EDS day 14 to 21, newly formed spindle-shaped progenitor Leydig cells appear near the peritubular myoid cells [10]. Progenitor Leydig cells began to possess biomarkers, including LHCGR, scavenger receptor class B member 1 (SCARB1), steroidogenic acute regulatory protein (STAR), cytochrome P450 cholesterol side chain cleavage (CYP11A1), HSD3B1, and 17α-hydroxylase/17,20-lyase (CYP17A1, 3, 9, 10]. Progenitor Leydig cells did not possess the last-step androgen biosynthetic enzyme 17β-hydroxysteroid dehydrogenase 3 (HSD17B3) and a glucocorticoid-metabolizing enzyme11β-hydroxysteroid dehydrogenase 1 (HSD11B1) [9]. On post-EDS day 28, ovoid immature Leydig cells emerge, with the expression of HSD17B3 and HSD11B1 [9], and on post-EDS day 56, polyhedral adult Leydig cells appear with a full capacity of testosterone secretion [11, 12]. Interestingly, the developmental process can also be mimicked in vitro using a Leydig cell-free seminiferous tubule (ST) culture system, in which stem Leydig cells on the surface of STs can be induced to commit into the Leydig cell lineage and to experience the transition from progenitor into immature and finally into adult Leydig cells with capability of producing testosterone. These STs was cultured in the Leydig cell differentiation-inducting medium (DIM), which was prepared by adding insulin-transferrin-selenium (ITS), LH, and lithium chloride (Li) to DMEM/F12 (1:1, v/v) medium [13, 14].

In the current study, we used both in vivo EDS-treated Leydig cell regeneration model and in vitro ST culture system to address the effects of MCP-1 on the Leydig cell development.

## Results

### MCP-1 increases testosterone levels in vivo

**Fig. 1:**
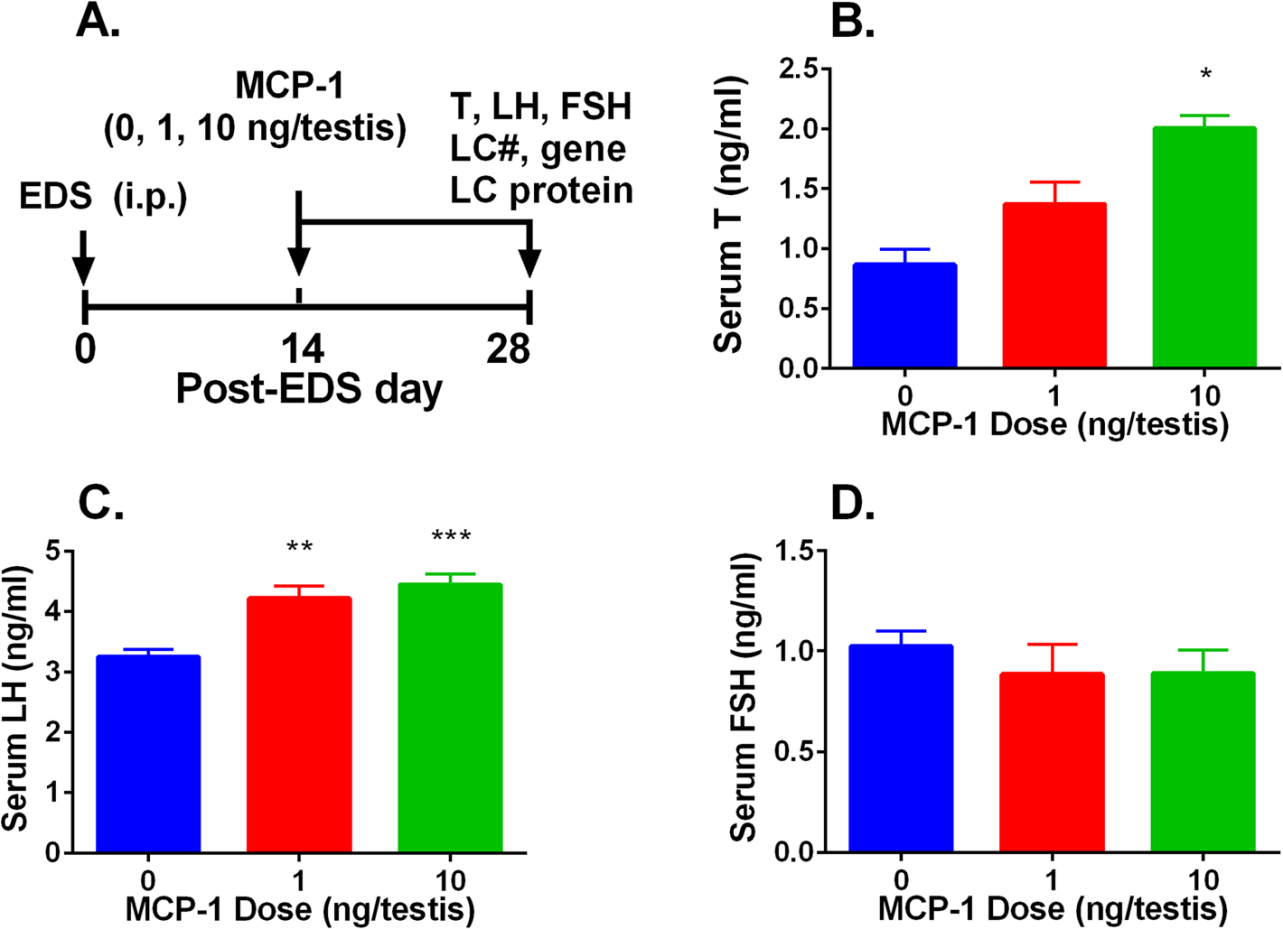
MCP-1 affects serum testosterone, LH, FSH levels in vivo. **a** Regimen of the animal experiment: LC, Leydig cells; #, cell number. Rats were treated with MCP-1 for 14 d. **b** Serum testosterone (T) levels. **c** LH levels. **d** FSH levels. Mean ± SEM, *n* = 6, *P < 0.05, **P < 0.01, and ***P < 0.001 designate significant difference when compared to the control (0 ng/testis MCP-1)

We used an in vivo Leydig cell regeneration model in rats to study the effects of MCP-1. MCP-1 (0, 1, or 10 ng/testis/day) was injected to rats for 14 d (Fig. 1a). MCP-1 significantly increased serum testosterone levels at 10 ng/testis (Fig. 1b) and LH levels at 1 and 10 ng/testis (Fig. 1c). However, it did not alter serum FSH levels (Fig. 1d). These results indicate that MCP-1 promotes Leydig cell regeneration process.

### MCP-1 has no effects on Leydig cell number in vivo

**Fig. 2:**
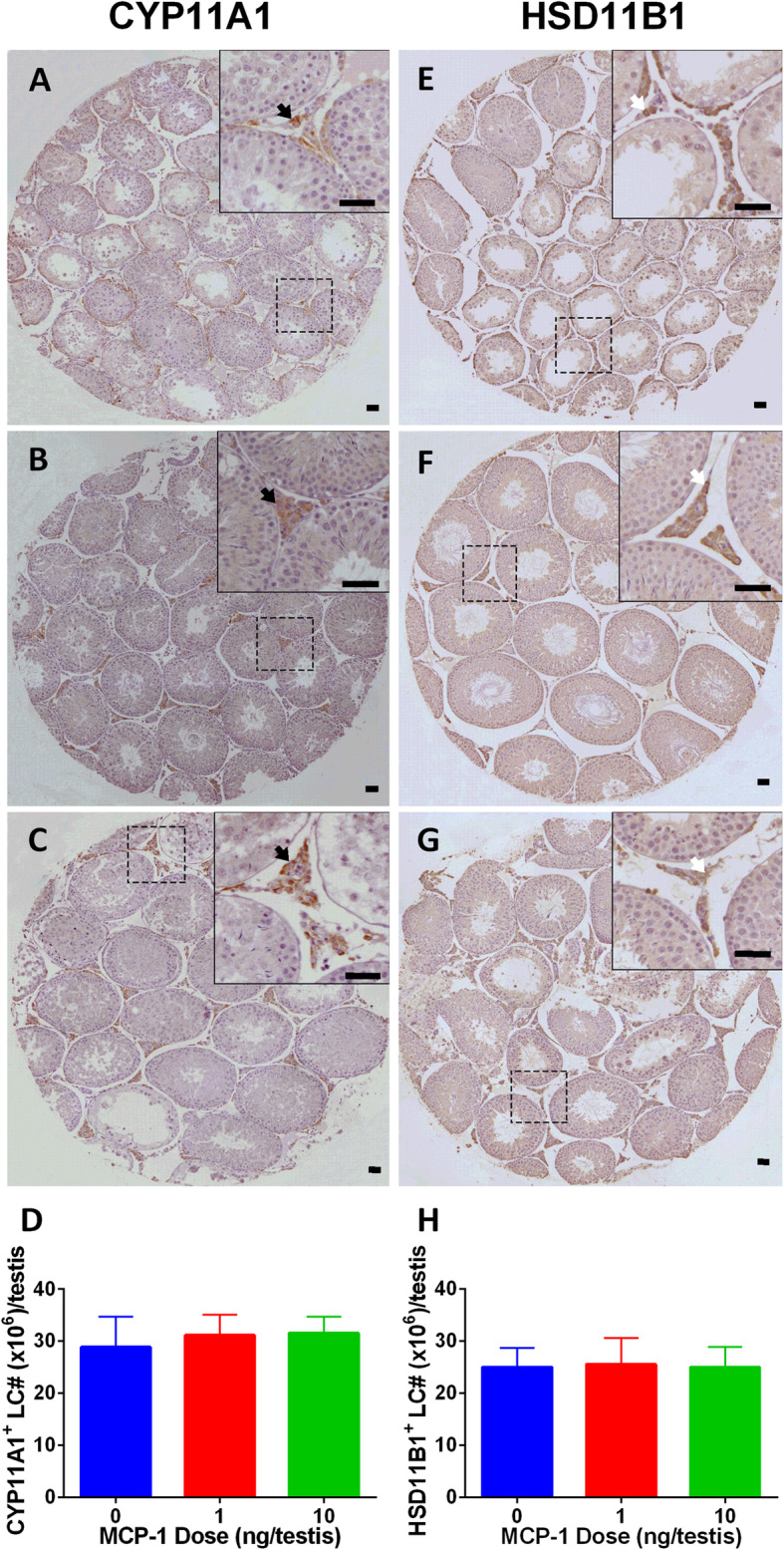
Morphology of Leydig cells in the testes after in vivo MCP-1 treatment. Immunohistochemistry of CYP11A1 (**a**-**c**) and HSD11B1 (**e**-**g**) of the testes from the rats treated with 0, 1, and 10 ng/testis MCP-1 from the 14th day after EDS. Black arrow shows CYP11A1 positive Leydig cells. White arrow designates HSD11B1 positive Leydig cells. Bar = 50 μm. **a** and **e**: the control (0 ng/testis MCP-1); **b** and **f**: (1 ng/ testis MCP-1); **c** and **g**: (10 ng/testis MCP-1); **d** and **h**: quantitative data. Mean ± SEM, *n* = 4–6. No significant difference between any two groups was observed

We adopted two biomarkers to identify Leydig cells: 1) CYP11A1, representing all Leydig cells; 2) HSD11B1, representing Leydig cells at the immature stage and beyond [9]. The numbers of CYP11A1-positive cells (Fig. 2a-d) and HSD11B1-positive cells (Fig. 2e-h) were not altered, indicating that the proliferation of Leydig cell precursors was not affected by MCP-1. We also used SOX9 to identify Sertoli cells (Supplementary Fig. S1) and found that MCP-1 did not alter Sertoli cell number either.

### MCP-1 regulates Leydig cell gene expression in vivo

**Fig. 3:**
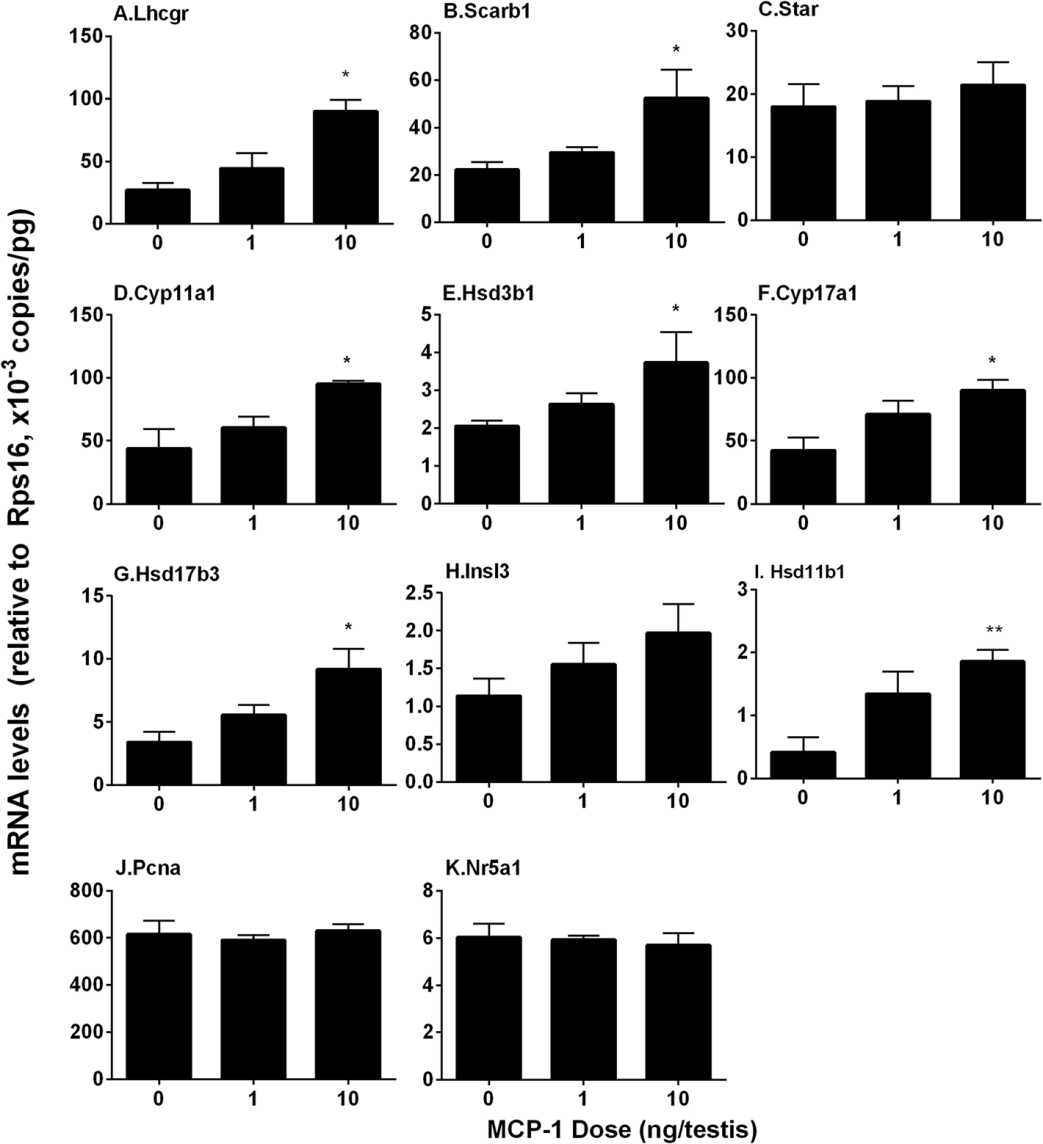
MCP-1regulates the expression of Leydig cell-specific genes in vivo. The mRNA levels of *Lhcgr*, *Scarb1*, *Star*, *Cyp11a1*, *Hsd3b1*, *Cyp17a1*, *Hsd17b3*, *Insl3*, and *Nr5a1* in the testes from the rats treated with 0, 1, and 10 ng/testis MCP-1 from the 14th day to 28th day after EDS were analyzed by qPCR. Mean ± SEM, *n* = 6, *P < 0.05 designates significant difference when compared to the control

The mRNA levels of Leydig cellspecific genes, *Lhcgr*, *Scarb1*, *Star*, *Cyp11a1*, *Hsd3b1*, *Cyp17a1*, *Hsd17b3*, *Hsd11b1*, *Insl3*, and *Nr5a1*, were measured by qPCR. The expression of *Lhcgr*, *Scarb1*, *Cyp11a1*, *Hsd3b1*, *Cyp17a1*, *Hsd17b3,* and *Hsd11b1* was significantly up-regulated by 10 ng/testis MCP-1 (Fig. 3a, b, d, e, f, and g) when compared to the control. *Pcna* level was not altered by MCP-1. We also calculated their levels after adjustment of CYP11A1 positive (Leydig) cells and found that their levels had similar changes (data not shown). This indicates that MCP-1 promotes Leydig cell regeneration at 10 ng/testis dose.

### MCP-1 regulates Leydig cell protein expression in vivo

**Fig. 4:**
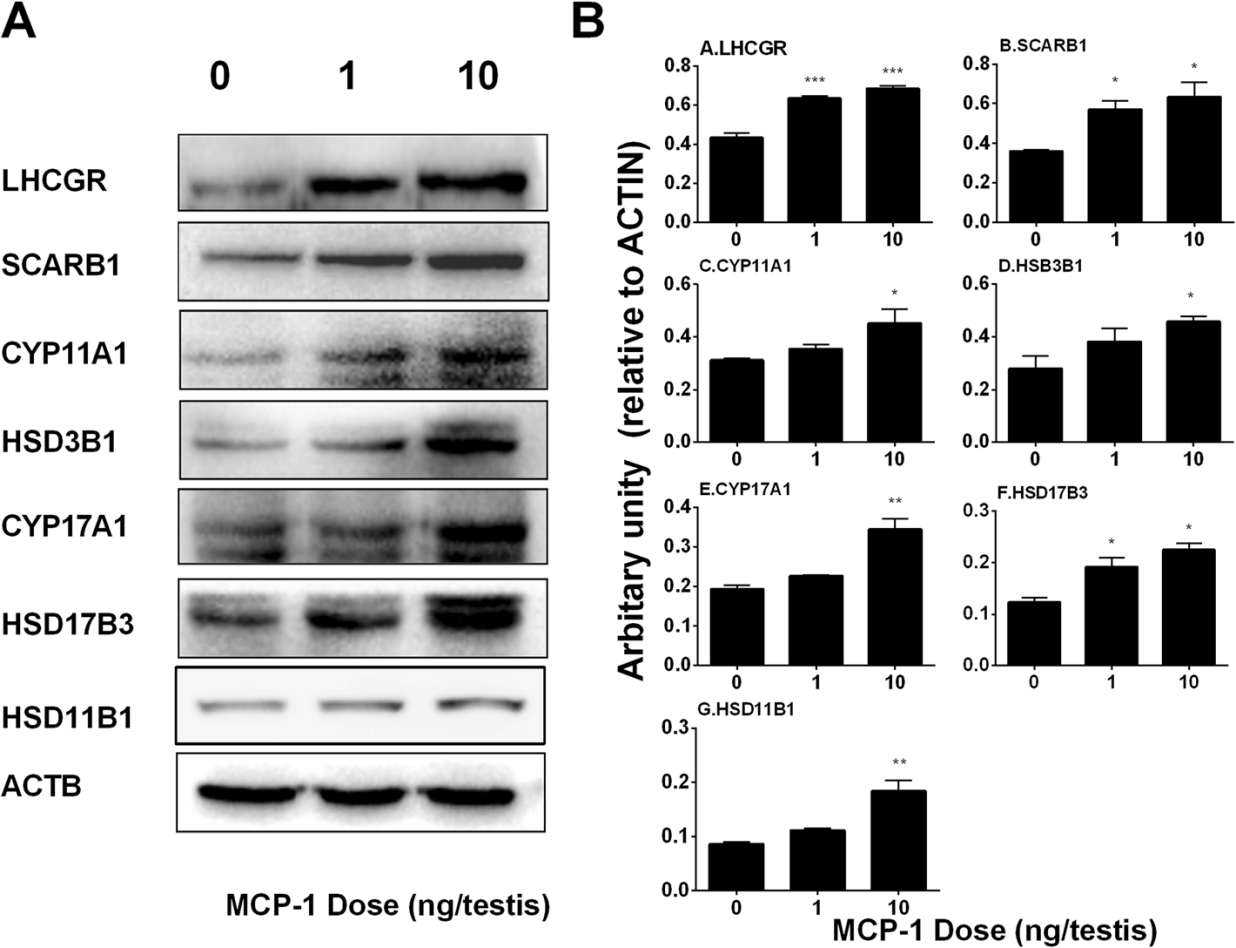
Effects of MCP-1 on Leydig cell-specific protein levels in vivo. Left panel: gel; Right panel: quantitative data. The protein levels of LHCGR, SCARB1, CYP11A1, HSD3B1, CYP17A1, HSD17B3, and ACTB (control) in the testes from rats treated with 0, 1, and 10 ng/testis MCP-1 from the 14th day to 28th day were analyzed by Western blot. Three testes each group were randomly selected. Mean ± SEM, *n* = 3, *P < 0.05, **P < 0.01, and ***P < 0.001 designate significant difference when compared to the control

As shown in Fig. 4, LHCGR, SCARB1, and HSD17B3 levels were significantly increased by MCP-1 at doses of ≥1 ng/testis and those of CYP11A1, HSD3B1, HSD11B1, and CYP17A1 were significantly increased at 10 ng/testis. We also calculated their levels after adjustment of CYP11A1 positive (Leydig) cells and found that their levels had similar changes (data not shown). This indicates that MCP-1 promotes the steroidogenic capacity of Leydig cells per se.

Furthermore, a semi-quantitative measurement of CYP11A1 and HSD11B1 levels in the individual Leydig cell was performed. It was found that MCP-1 (10 ng/testis) increased CYP11A1 and HSD11B1 densities in the Leydig cell, confirming the Western blotting data (Supplementary Fig. S2).

**Fig. 5:**
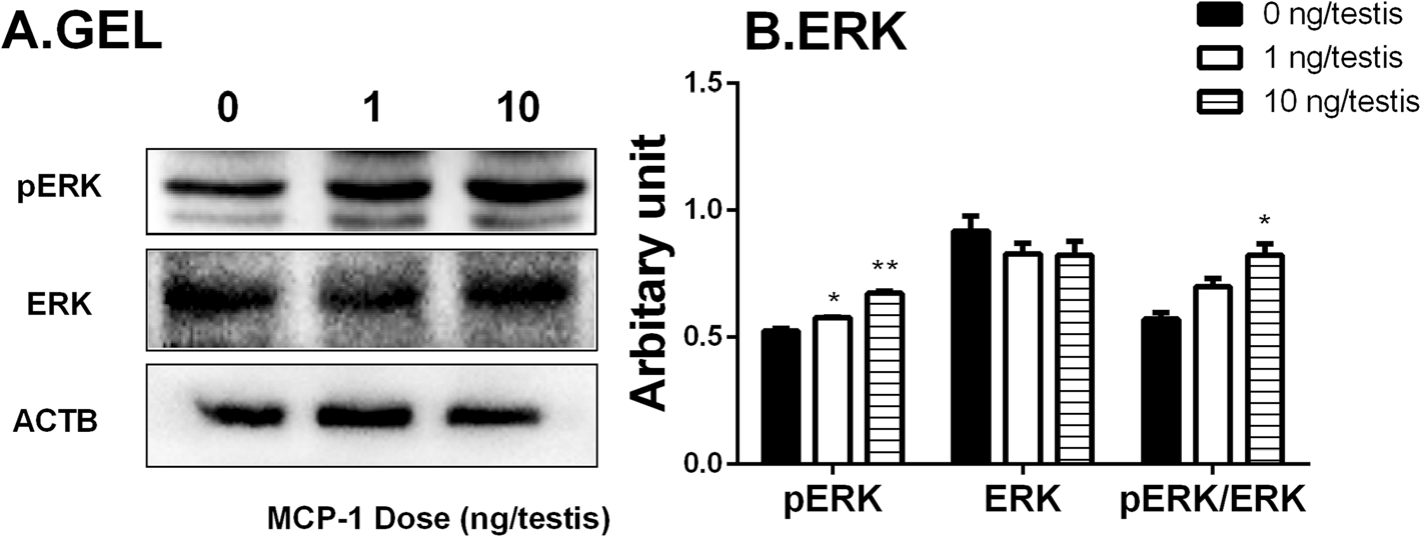
Effects of MCP-1 on EKR1/2 and its phosphorylation in vivo. Left panel: gel; Right panel: quantitative data. The protein levels of phosphorylated ERK1/2 (pERK), ERK1/2 (ERK), and the pERK/ERK ratio in the testes from rats treated with 0, 1, and 10 ng/testis MCP-1 from the 14th day to 28th day were analyzed by Western blot. Three testes each group were randomly selected. Their levels were adjusted to β-actin (ACTB). Mean ± SEM, *n* = 3, *P < 0.05, **P < 0.01 designate significant difference when compared to the control

Many studies have demonstrated that ERK1/2 pathway took part in the development of Leydig cells [15–17]. The downstream signals of MCP-1 in the testis were investigated. MCP-1 significantly increased phosphorylation of ERK1/2 (pERK1/2) without affecting the ERK1/2 levels, thus increasing the ratio of pERK1/2 to ERK1/2 in the MCP-1 treated testis (Fig. 5). These results indicate that the ERK1/2 pathway is involved in the MCP-1 mediated stimulation of Leydig cell development.

### MCP-1 has no effects on Leydig cell proliferation in vivo

**Fig. 6:**
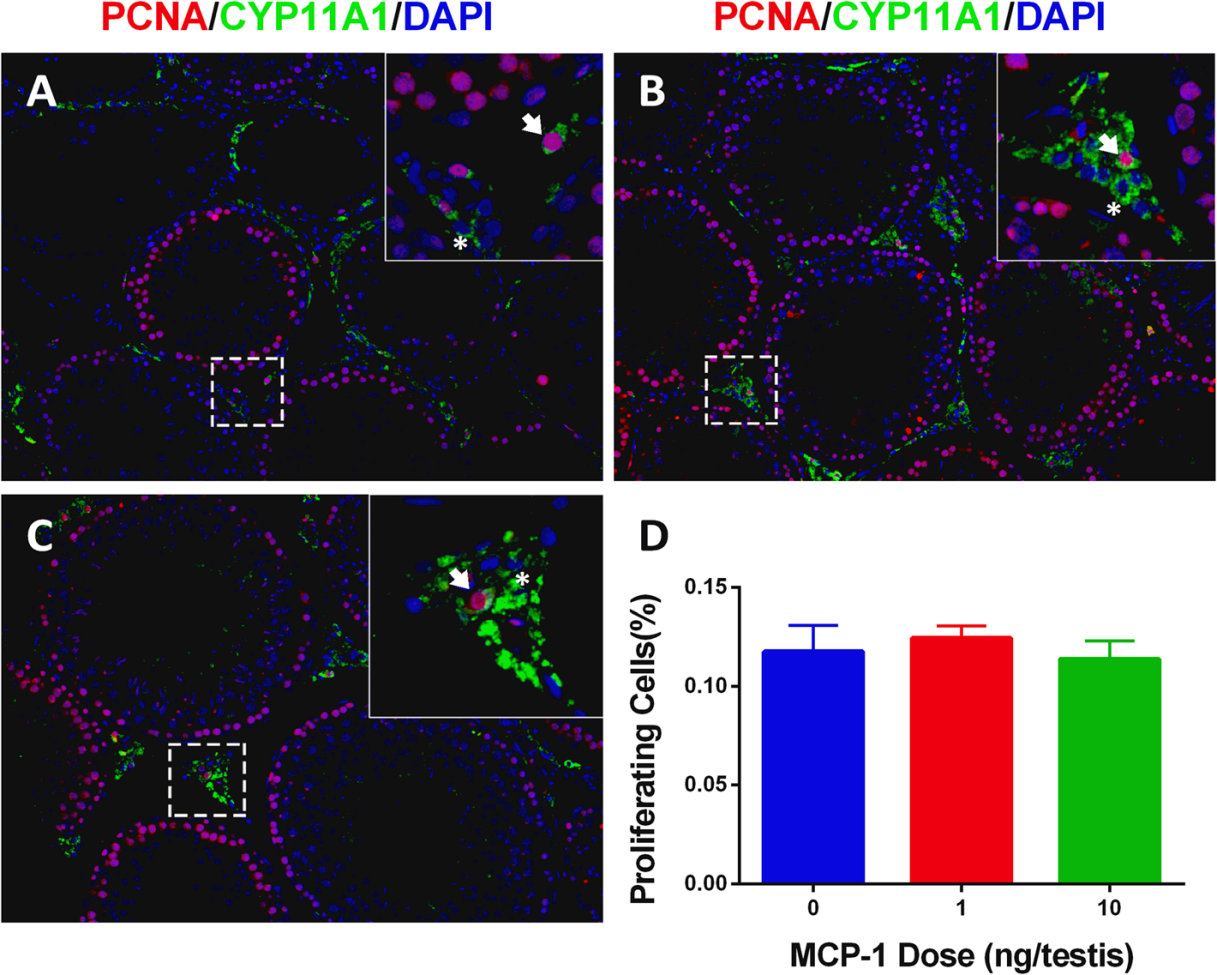
Effects of MCP-1 on the number of PCNA-positive Leydig cells in vivo. Immunofluorescent staining of PCNA (red color in the nucleus) and CYP11A1 (green color in the cytoplasm) and DAPI (blue color in the nucleus) as the contrasting staining of the sections of testes from rats treated with 0 (**a**), 1 (**b**), 10 (**c**) ng/testis MCP-1 from the 14th day to 28th day **d**: quantitative data. White arrow indicates PCNA-positive Leydig cells. White “*” indicates PCNA-negative Leydig cells. Mean ± SEM, *n* = 6. No significance was observed between any two groups.

Progenitor and immature Leydig cells have the capacity of cell division (Ge and Hardy, 1997). Double staining of PCNA and CYP11A1 was performed to judge Leydig cell proliferation. As shown in Fig. 6, MCP-1 had no effects on the percentage of PCNA-positive Leydig cells, confirming the unchanged Leydig cell number after MCP-1 treatment.

### MCP-1 has no effects on stem Leydig cell proliferation in vitro

**Fig. 7:**
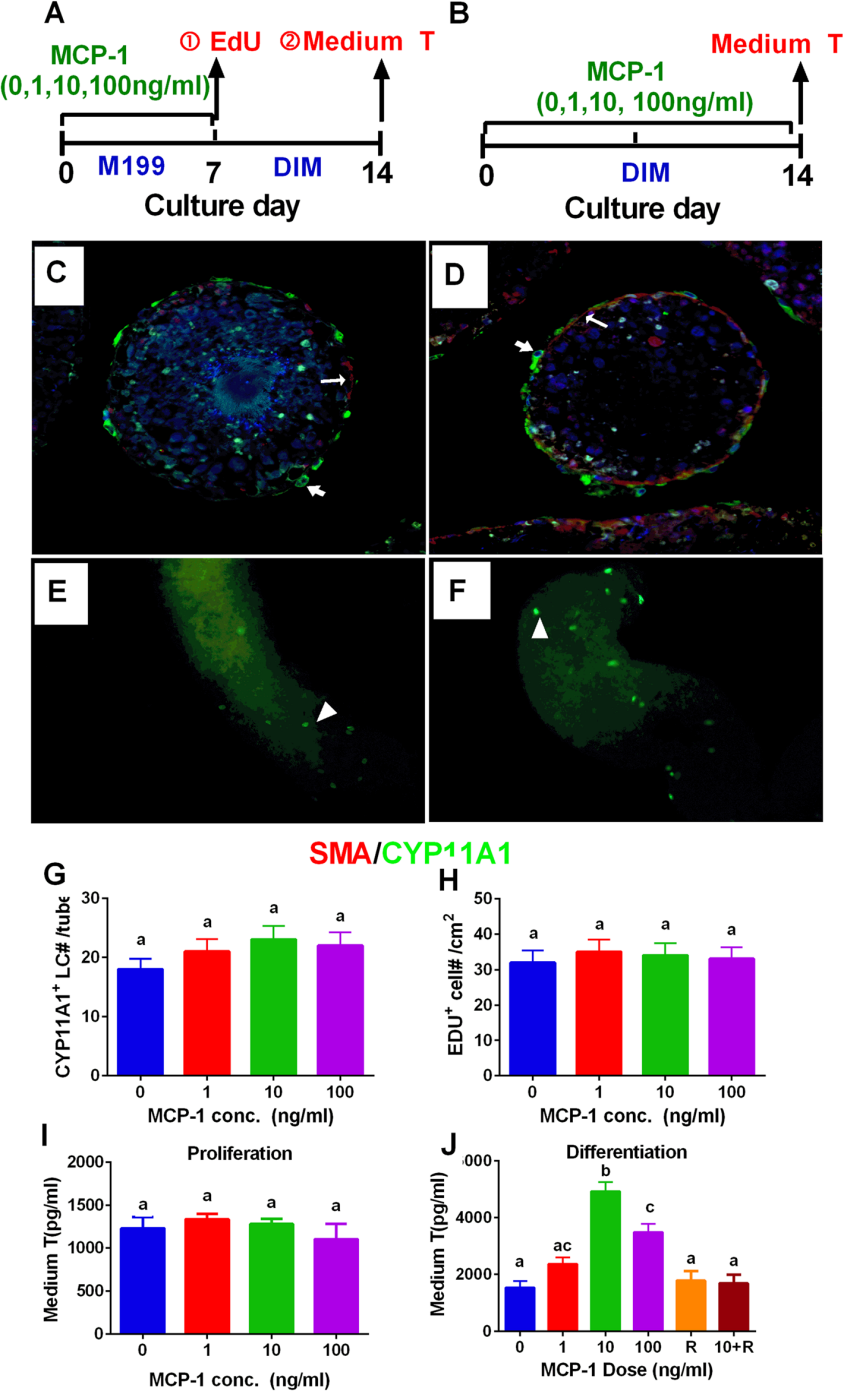
Effects of MCP-1 on stem Leydig cell proliferation and differentiation in vitro. **a**: regimen for stem Leydig cell (SLC) proliferation. Seminiferous tubules (STs) were cultured in MCP-1 (1–100 ng/ml) in M199 for 7 d and EdU incorporation for direct proliferation assay was conducted (① step) and the rest STs switched in the Leydig cell (LC) differentiation-inducing medium (DIM) for additional 7 d to induce SLCs to differentiate into LCs to secrete testosterone (T) into medium for indirect proliferation assay (② step) was conducted. **b**: regimen for SLC differentiation. STs were cultured in MCP-1 (1–100 ng/ml) in DIM for 14 d to induce SLCs to differentiate into LCs to secrete T into medium for differentiation assay was conducted. **c** and **d**: immunofluorescent staining of ST cross-section after MCP-1 (0 and 100 ng/ml) treatment from d7 to 14 of culture in DIM for 7 d (in the indirect proliferation assay), respectively. ST cross-sections with CYP11A1 staining (green color, thick arrow) showed the formation of LCs. α-smooth muscle actin (SMA) staining (red color, thin arrow) showed the peritubular myoid cells, which circled the STs. CYP11A1-positive cells were outside the SMA-positive cells, indicating that they were differentiated from SLCs on the surface of the tubules. **e** and **f**: immunofluorescent staining for EdU-incorporated SLCs (white arrowhead) on the surface of STs after MCP-1 (0 and 100 ng/ml) treatment from d1 to 7 of culture for 7 d (in the direct proliferation assay), respectively. **g**: quantitative data of LC number per cross section (for **c** and **d**), mean ± SEM, *n* = 6; **h**: quantitative data of EdU-positive SLC number per cm^2^ (for **e** and **f**), mean ± SEM, *n* = 6; **i**: quantitative data of T levels in indirect proliferation assay in the ② step in **a**, mean ± SEM, *n* = 6. **j**: quantitative data of T levels after 7-d MCP-1(0–100 ng/ml) and/or RS102895 treatment, R = 0.1 μM RS102895 from d7 to 14 of culture. Mean ± SEM, *n* = 6. The identical letters showed no significant difference between two groups at *P* < 0.05

Leydig cell-depleted ST culture system was used to address this issue. Stem Leydig cells reside on the surface of STs [13]. We treated STs at 0, 1, 10, and 100 ng/ml for 7 d in M199 medium and then added EdU to investigate stem Leydig cell proliferation (Fig. 7a). We did not find the change in the labeling index of stem Leydig cells (Fig. 7e, f, h), indicating that MCP-1 has no effects on stem Leydig cell proliferation. Furthermore, we used an indirect method by treating STs with MCP-1 for 7 d and then switching these STs in the DIM for additional 7 d (Fig. 1a). MCP-1 did not increase medium testosterone levels either (Fig. 7i), further confirming the above assertion. STs were cultured with MCP-1 (0, 1, 10, 100 ng/ml) in DIM for 7 d. Peritubular myoid cells of STs were labeled with SMA (red color) and Leydig cells were labeled with CYP11A1 (green color). As shown in Fig. 7c and d, MCP-1 had no effects on the number of Leydig cells after differentiation from stem cells (Fig. 7g), suggesting that MCP-1 had no effect on the self-renewal of the stem Leydig cells.

### MCP-1 stimulates stem Leydig cell differentiation in vitro

In order to investigate the effects of MCP-1 on the differentiation of stem Leydig cells, we cultured STs in DIM with or without MCP-1 for 14 d. MCP-1 significantly increased medium testosterone levels (Fig. 7j), with 10 ng/ml MCP-1 reaching the maximal response. This suggests that MCP-1 promotes stem Leydig cell differentiation. To further dissect MCP-1 mediated signaling pathway, a selective CCR2 receptor antagonist, RS102895, was used alone or in combination with MCP-1. RS102895 alone did not affect medium testosterone levels but significantly reversed MCP-1 induced increase of testosterone levels (Fig. 7j). This indicates that MCP-1 promotes stem Leydig cell differentiation via CCR2 receptor.

### MCP-1Regulates Leydig cell gene and Protein expression in vitro

**Fig. 8:**
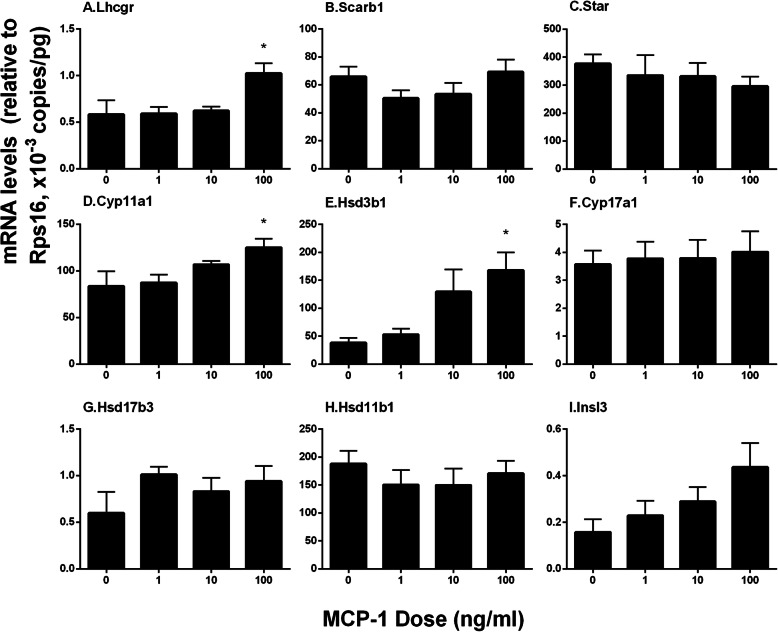
MCP-1 up-regulates Leydig cell gene expression in vitro. Seminiferous tubules were treated with MCP-1 (0, 1, 10, and 100 ng/mL) from day 7 to 14 of culture. **a**-**i**: the mRNA levels of *Lhcgr*, *Scarb1*, *Star*, *Cyp11a1*, *Hsd3b1*, *Cyp17a1*, *Hsd17b3*, *Hsd11b1*, and *Insl3*. *Rsp16* served as the internal control. Mean ± SEM, *n* = 6, *P < 0.05 designates significant difference when compared to the control

**Fig. 9:**
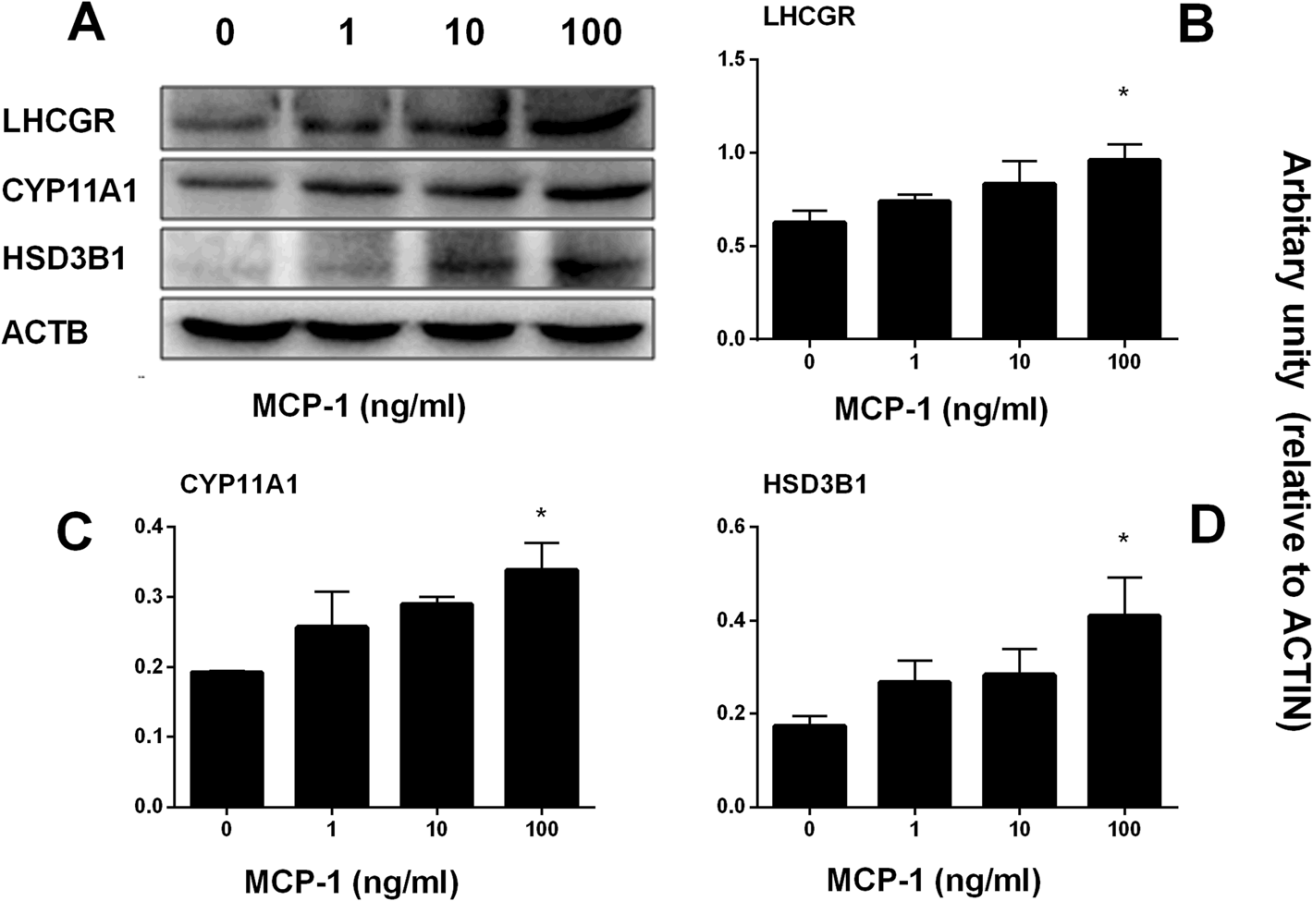
MCP-1 affects Leydig cell-specific protein levels in vitro*.* Left panel: gel; Right panel: quantitative data. The protein levels of LHCGR, CYP11A1, HSD3B1, and ACTB (control) were analyzed by Western blot in the testes from the seminiferous tubules treated with 0, 1, 10 and 100 ng/ml MCP-1 from day 7 to 14 of culture. Mean ± SEM, *n* = 6, * *P* < 0.05 designates significant difference when compared to the control

Leydig cell specific gene and protein expression levels in STs after MCP-1 treatment were measured. MCP-1 increased the mRNA levels of *Lhcgr*, *Cyp11a1* and *Hsd3b1* at 100 ng/ml dose (Fig. 8), while it did not affect those of *Scarb1*, *Star*, *Cyp17a1*, *Hsd17b3*, *Hsd11b1*, and *Insl3*. Indeed, Western blot confirmed that MCP-1 significantly increased the levels of LHCGR, CYP11A1, and HSD3B1 at 100 ng/ml dose (Fig. 9). These results suggest that MCP-1 promotes stem Leydig cell differentiation by up-regulating *Lhcgr*, *Cyp11a1* and *Hsd3b1* expression.

## Discussion

MCP-1 (CCL2) is a member of the low-molecular-weight cytokine family with chemotactic activity. MCP-1 selectively binds to the surface receptor CCR2, which is coupled to heterotrimeric G protein for signal transduction. Although it has been shown that MCP-1 can play many roles, such as stimulating immune cell recruitment, regulating immune cell trafficking, and inducing inflammatory responses [5], we are the first to identify the critical role of MCP-1 in regulating the regeneration of stem Leydig cells in the current study.

In the testis, MCP-1 is constitutively expressed in peritubular myoid cells [6] as well as stem Leydig cells and the cells in the Leydig cell lineage [4]. Testicular peritubular myoid cells also express MCP-1 and several cytokines such as interleukin-1 and tumor necrosis factor-α (TNF-α) that can dramatically upregulate expression of MCP-1 in vitro [6]. In the experimental autoimmune orchitis of rats, MCP-1 expression was increased in Leydig and peritubular, mononuclear, and endothelial cells and also detected in Sertoli cells in severe orchitis [7]. This suggests that MCP-1 might regulate stem Leydig cell development via both paracrine and autocrine pathways in both physiological and inflammatory conditions. According to a previous study [18], the concentration of MCP-1 in the intratesticular fluid of normal rat testis was 38 ng/ml and its levels were increased by 20 to 400 folds after LPS treatment. Therefore, the concentrations of MCP-1 were within normal physiological range in vivo study. In the inflammatory condition, its level will reach over 15,000 ng/ml. Indeed, in the current study, we demonstrated that MCP-1 promoted Leydig cell regeneration by inducing their differentiation, as evidence of the facts that MCP-1 increased the expression of Leydig cell specific genes, such as *Lhcgr*, *Scarb1*, *Cyp11a1*, *Hsd3b1*, *Cyp17a1*, and *Hsd17b3* and their proteins (LHCGR, SCARB1, CYP11A1, HSD3B1, CYP17A1, and HSD17B3) as well as increased serum testosterone levels in EDS-treated Leydig cell regeneration model in rats.

The end of in vivo rat experiment was set on post-EDS day 28, when the type of Leydig cells regenerated is immature Leydig cells as reported in our previous study [9]. CYP11A1-positive cells represent all cells in the Leydig cell lineage (including progenitor and immature Leydig cells) in the current study. HSD11B1-positive cells represent immature Leydig cells in the current study. Since the number of both CYP11A1-positive and HSD11B1-positive cells remain unchanged (Fig. 2), this suggests that the number of progenitor and immature Leydig cells is not changed after MCP-1 treatment. In the previous study, we demonstrated that on post-EDS day 28 all Leydig cells in the control group were immature Leydig cells [9]. Therefore, the source of elevating T level should come from the increasingcapacity of Leydig cells to secrete T rather than from the increasing number of immature Leydig cells.

We also performed qPCR and Western blot to measure CYP11A1, HSD3B1, CYP17A1, and HSD17B3 and their gene expression levels (Figs. 3 and 4) and calculated them after adjustment to the CYP11A1-positive cells and again we showed that their levels were significantly increased after MCP-1 treatment. This suggests that MCP-1 promotes the capacity of steroidogenic enzymes because the Leydig cell number was not changed.

We did not perform immunohistochemical staining of HSD3B1, CYP17A1, and HSD17B3 and calculated HSD3B1, CYP17A1, and HSD17B3 positive cells. When HSD3B1 antibody was used, there was high background non-specific staining and the calculation of HSD3B1 positive cells could cause misleading data. Therefore, we did not use the HSD3B1 data in the current study. CYP17A1 and HSD17B3 antibodies were good for Western blotting but were not suitable for immunohistochemical staining. Therefore, we could not detect CYP17A1 and HSD17B3 positive cells.

Apparently, MCP-1 in vivo also increased LH secretion in the pituitary as shown by the increase of its levels in serum. Although MCP-1 was injected intratesticularly, the increase of pituitary LH secretion could be due to the entrance of MCP-1 into the blood system after injection. However, how MCP-1 regulates the secretion of LH is not clear. In the present study we did not evaluate the effects of MCP-1 on gonadotroph cell function for LH release. This requires further investigation.

The effects of MCP-1 to promote the differentiation of stem Leydig cells in DIM (containing LH and lithium chloride) might also be exerted locally. Our previous study showed in the in vitro ST culture system LH is essential for inducing the appearance of adult Leydig cells and secretion of testosterone [3]. Recently, a modified culture medium called DIM (containing LH and lithium chloride) was used to speed up the differentiation of stem Leydig cells into the Leydig cell lineage and shorten the culture 28-day culture period in LH-containing medium into 14-day DIM [13]. Using the in vitro ST culture system in DIM, we clearly demonstrated that MCP-1 synergized DIM-induced medium testosterone levels and up-regulated Leydig cell specific gene (*Lhcgr*, *Cyp11a1*, and *Hsd3b1*) and their protein expression. Apparently, MCP-1 was less potent to induce the differentiation of stem Leydig cells in vitro, because MCP-1 up-regulate fewer genes in vitro (only *Lhcgr*, *Cyp11a1*, and *Hsd3b1*) than in vivo (*Lhcgr*, *Scarb1*, *Cyp11a1*, *Hsd3b1*, *Cyp17a1*, and *Hsd17b3*) and it required a concentration as high as 100 ng/ml for MCP-1 to up-regulate these three gene sexpression in vitro. Therefore, in vivo, MCP-1 possibly stimulated the differentiation of stem Leydig cells via acting both on pituitary to increase LH secretion and on stem Leydig cells directly.

Although MCP-1 has been reported to stimulate proliferation of many cells in the immune system and cancer cells [5], in the current study, we did not observe any effects of MCP-1 on the proliferation of Leydig cell precursors in vivo or in vitro.

Previous studies showed that in the EDS model Leydig cells underwent apoptosis during the first week [11, 12]. Two-four days after EDS there are many cytokines and inflammatory factors due to the death of Leydig cells were up-regulated [11, 12, 19] and there is the peak of peritubular/myoid cell proliferation possibly due to the increase of these factors. Therefore, it is not good time-point for the treatment of MCP-1 because it itself is one of these cytokines. Four days after EDS there are still a few Leydig cells left and 7 days after EDS all Leydig cells are depleted [20, 21]. Therefore, we started the injection of MCP-1 on post-EDS 14, when there are no CYP11A1 positive Leydig cells existing [20] and stem Leydig cells are ready to commit into Leydig cell lineage.

In the in vivo Leydig cell regeneration model, it was found that MCP-1 did not alter the number of both CYP11A1-positive or HSD11B1-positive cells (Fig. 2) and it did not change the percentage of PCNA-positive Leydig cells and altered Pcna mRNA level, suggesting that MCP-1 did not promote the proliferation of progenitor and immature Leydig cells in vivo. In the in vitro ST culture system, MCP-1 did not increase the EdU incorporation into stem Leydig cells, and it did not increase the number of adult Leydig cells after Leydig cell differentiation induction and medium testosterone levels after initial one-week treatment of MCP-1 in M199 medium and subsequent one-week Leydig cell differentiation induction in DIM.

The effects of MCP-1 on the differentiation of stem Leydig cells into the Leydig cell lineage might be mediated by CCR2 receptor, because the MCP-1 induced medium testosterone levels could be reversed by CCR2 antagonist, RS102895.

Although the exact downstream signaling of MCP-1 is unclear, the increase of ERK1/2 phosphorylation by MCP-1 could be one of possiblepathways. ERK1/2 signaling pathways has been reported to take part in Leydig cell development [15–17, 22]. Many factors including epidermal growth factor, insulin-like growth factor 1, and kit ligand have been found to upregulate ERK1/2 phosphorylation. The epidermal growth factor can bind epidermal growth factor receptorin the Leydig cell precursors, activating phosphorylation of ERK1/2 [16, 23, 24]. Epidermal growth factor receptor can partially mediate LHCGR-stimulated activation of ERK1/2 cascade in immature Leydig cells to increase androgen production [16, 23, 24]. Indeed, LH has been found to the major hormone to regulate Leydig cell differentiation after stem Leydig cells begin to commit into the Leydig cell lineage with the expression of LHCGR, because the knockout of *Lhcgr* in mice led to Leydig cell hypoplasia, with few adult Leydig cells existing in the interstitium of the testis [25, 26].

Altogether, the results indicated that ERK1/2 phosphorylation might be the signal pathway of MCP-1 in the regulation of Leydig cell differentiation or regeneration.

### Conclusion

MCP-1 stimulates the differentiation of stem Leydig cells without affecting their proliferation.

## Methods

### Chemicals and kits

MCP-1 was purchased from PeproTech (no. 96–300–04-20, Rocky Hill, NJ). RS102895 hydrochloride (RS), a CCR2 blocker, was purchased from Abcam Shanghai (no. ab120812, purity: > 99%, Shanghai, China). M199 and DMEM/F12 media were purchased from Invitrogen (Carlsbad, CA). EDS was obtained from Pterosaur Biotech Co (Hangzhou, China). Adult (60-day-old) male Sprague Dawley rats were purchased from Shanghai Experimental Animal Center (Shanghai, China).

### Animals and treatments

Rats were acclimated in the following conditions: 12 h dark/light cycle, 21–25 °C, 55% relative humidity, and ad libitum access to water and food. At the time of experiment, the median weight of rats was 381 g (315 g ~ 450 g) and the median age of rats was 67 d. After 7-d acclimation, each rat was given EDS (75 mg/kg body weight) via intraperitoneal injection [27]. Previous studies demonstrated that a single dose (75 mg/kg) of EDS killed all Leydig cells in the rat testis on the 7th day after EDS and Leydig cells were completely recovered 56 d later [3, 9, 28]. Eighteen rats were randomly divided into three groups with 6 rats per group: 0 (vehicle control), 1, and 10 ng/testis MCP-1 on the 7th day after EDS. According to a previous study [18], the concentration of MCP-1 in the intratesticular fluid of normal rat testis was 38 ng/ml and its levels were increased by 20 to 400 folds after LPS treatment. Therefore, we selected the low-dose range for MCP-1 administration. MCP-1 was dissolved in normal saline for testicular injection. Both testes of each rat daily received an aliquot (25 μl) of MCP-1 (0, 1, or 10 ng/testis) for 14 d, starting on the 14th day after EDS. Intratesticular administration of MCP-1 was adopted to avoid its possible systemic action on the hypothalamus and pituitary. No testicular damage was observed after daily intratesticular injections of 25 μl solution after a pathological examination of the injected testis in the pilot study. We selected the 14th day after EDS as the starting injection day of MCP-1 because stem Leydig cells begin to commit into the Leydig cell lineage [3]. On the 28th day after EDS, when immature Leydig cells emerge in the testis [29], rats were euthanized by asphyxiation with progressively increasing concentration of CO_2_ and the blood samples were collected. Sera were harvested for testosterone, FSH (follicle-stimulating hormone), and LH analysis. One testis per rat was collected for mRNA and protein expression studies. The contralateral testis was fixed in Bouin’s solution for immunohistochemical staining. The study was submitted to the Animal Care and Use Committee of Wenzhou Medical University, who approved this study (protocol number: wydw2016–0311).

### Assay of serum testosterone levels

As mentioned previously [30], the serum testosterone concentration was measured using the IMMULITE 2000 Total Testosterone kit (Siemens, Germany). Two samples from each rat were used for testing. The negative control and positive control were blank charcoal-stripped rat serum and normal male adult serum (set at 2 ng/ml), respectively. The minimum detective concentration of testosterone is 0.2 ng/ml. Normal adult male rat serum was used as an internal control. The inter-assay and intra-assay variances were within 15%.

### Determination of serum LH and FSH levels

As mentioned previously [30], serum LH and FSH levels were measured using the ELISA kits (Chemicon, CA). Briefly, serum samples were poured in duplicate into pre-coated 96-well plates and incubated for 2 h, followed by peroxidase-conjugated IgG anti-LH or anti-FSH solution. The negative control and positive control were blank medium and normal male adult serum, respectively. Substrates were added and the quantification of LH or FSH levels was obtained by a microplate reader. Two samples per animal were used for detection. The intra-assay variances of the assays for LH were 8.9% and for FSH 18.3%.

### Isolation and culture of STs

STs were isolated as previously described [31]. In brief, adult male rats were treated with EDS above and testes were collected 7 d later and STs were mechanically separated and testes of three rats were put together. ST parts were cut into about 1 cm in the length and equally distributed in M199. STs were cultured in DIM in 5% CO_2_ at 34 °C for 14 d. Stem Leydig cells on the surface of the STs were differentiated into adult Leydig cells, which secrete testosterone into the medium [13]. STs were treated with different concentrations of MCP-1 and its inhibitor (RS102895) in the following experiments. Six to twelve isolations were performed.

### Assay of stem Leydig cell proliferation

The proliferation of stem Leydig cells was evaluated as previously described [13]. In brief, STs from three rats were cultured in M199 with 1–100 ng/ml MCP-1 for 7 d as above, then they were divided into two parts: one part was treated with Click-iT® EdU (EdU) Alexa Fluor® 488 Imaging Kit (Life Technologies, OR) and the other part was switched to DIM for additional 7 d, as an indirect measurement of the proliferation of stem Leydig cells. Media were collected for the detection of testosterone concentrations.

### Determination of EdU incorporation into stem Leydig cells

EdU incorporation into stem Leydig cells was conducted as previously described [13]. After cultured in EdU-containing M199 medium for 24 h, STs were dipped in 4% paraformaldehyde for fixation and reacted with the reaction solution. A fluorescent microscope (Olympus, Tokyo, Japan) was used to snap EdU-positive stem Leydig cells. Using Image-Pro plus 6.0 (Media Cybernetics, Inc., Rockville, MD), EdU positive cells were counted and calculated by the flat surface area of ST.

### Assay of stem Leydig cell differentiation in vitro

The differentiation of stem Leydig cells was evaluated as previously described [13]. In brief, STs were cultured in DIM in combination with 1–100 ng/ml MCP-1 for 7–14 *D. media* were collected for the detection of testosterone concentrations.

### Real-time PCR (qPCR)

Total RNAs were purified from testes and STs using Trizol kit (Invitrogen, Carlsbad, CA) as previously described [27]. RNA concentration measurement, cDNA synthesis and qPCR with SYBR Green qPCR Kit (Roche, Basel, Switzerland) were conducted as previously described [13]. The mRNA levels of Leydig cell specific genes, *Lhcgr*, *Scarb1*, *Star*, *Cyp11a1*, *Hsd3b1*, *Cyp17a1*, *Hsd17b3*, *Hsd11b1*, *Insl3,* and *Nr5a1* were determined. The primer list was provided in Supplementary Table S1. The mRNA levels were calculated by a standard curve. A Ct value and a logarithm of a series of diluted standards were used to create a standard linear equation, and the target mRNA was calculated from the measured Ct value and normalized to *Rps16* (internal control). Intra-assay variances of the assays for qPCR of the mRNAs were within 20%.

### Western blot analysis

Western blotting was carried out as previously described [27]. Briefly, the testis or STs were lysed in RIPA lysis buffer (Bocai Biotechnology, China) and homogenized. The protein concentrations of samples were measured by BCA™ Protein Assay Kit (Takara, Japan). An aliquot (30 μg) of proteins was denatured, loaded, electrophoresed and transferred onto the nitrocellulose membrane. The membrane was blocked for non-specific binding and incubated with primary antibodies: LHCGR, SCARB1, CYP11A1, HSD3B1, CYP17A1, HSD17B3, pERK1/2, ERK1/2, and ACTB (β-actin, the house-keeping protein as the internal control). The antibody list was provided in Supplementary Table S2. The membrane was blotted with HRP-conjugated secondary antibody (Bioword, Louis Park, MN) and protein bands were visualized with Super-Signal West Pico chemiluminescent substrate (Pierce Biotechnology, Radford, IL) by Universal Hood II (BioRad, Hercules, CA). The density of the target band was calculated using ImageJ software and the protein levels were adjusted to ACTB. The intra-assay variances of the Western blotting assays for protein levels were within 20%.

### Immunohistochemistry and immunofluorescence

Testes and STs were embedded in paraffin, and sectioned (6 μm) as previously described [13, 32]. For immunohistochemistry, sections were stained with the antibodies of CYP11A1 [a general biomarker of Leydig cells [9]], HSD11B1 [the biomarker for immature and mature Leydig cells [9, 33]] and SOX9 [the biomarker for Sertoli cells [34]]. Diaminobenzidine was used as substrate. Immunofluorescence staining of testis section was performed as previously described [35]. The primary antibodies of CYP11A1, PCNA (the general biomarker of proliferating cells), and α-smooth muscle actin (SMA, a general biomarker of peritubular myoid cells) were added, followed by fluorescent secondary antibody (Alexa-conjugated anti-rabbit or anti-mouse IgG, 1:500). Negative control was rabbit IgG. Isotype IgG controls were served as the control. These actions were counterstained with DAPI.

### Stereological analysis of Leydig cells and Sertoli cells

Stereological analysis was performed as previously described [36]. CYP11A1 and HSD11B1 were used to identify Leydig cells and SOX9 was used to identify Sertoli cells. Sampling of the testis was performed according to a fractionator technique as previously described [36]. Cell number estimation is to count cells on the selected set of paraffin sections that were obtained from the sequential sampling steps of the testis. The intra-assay variances of the assays for Leydig cell and Sertoli cell numbers were within 21%.

### Semi-quantitative Immunohistochemical staining of Leydig cell proteins

CYP11A1 and HSD11B1 are two important steroidogenic enzymes in the Leydig cells [1]. Immunohistochemical staining of CYP11A1 and HSD11B1 in the testis sections was performed as above. As previously described [32], the CYP11A1 and HSD11B1 density were determined semi-quantitatively. Briefly, the color density and background of CYP11A1 or HSD11B1 were measured by Image-Pro Plus (Media Cybernetics, Silver Spring, MD). The net density of CYP11A1 or HSD11B1 was calculated. Leydig cells were evaluated in each testis section and were averaged as one sample. The intra-assay variances of the assays for CYP11A1 and HSD11B1 densities were within 15%.

### Statistical analysis

Data were presented as the mean ± SEM. *P* < 0.05 was designated as statistically significant after statistical analysis. The differences of groups were evaluated by one-way ANOVA followed by ad hoc Turkey’s multiple comparisons to compare between two groups using GraphPad software (version 6).

## Supplementary information

**Additional file 1: Supplementary Figure S1.** Morphology of Sertoli cells in the testes after in vivo MCP-1 treatment. Immunohistochemical staining of SOX9 (Panels A-C) of the testes from the rats treated with 0, 1, and 10 ng/testis MCP-1 from post-EDS day 14 for 14 days. Black arrow designatesSOX9 positive (brown color in the nucleus) Sertoli cells. Bar = 50 mm. Panel A, the control (0 ng/testis MCP-1); Panel B, 1 ng/ testis MCP-1; Panel C, 10 ng/testis MCP-1; Panel D, quantitative data. Mean ± SEM, *n* = 4–6. No significant difference was observed.

**Additional file 2: Supplementary Figure S2.** Semi-quantitative measurement of CYP11A1 and HSD11B1 levels of the Leydig cells after in vivo MCP-1 treatment. Immunohistochemical staining of CYP11A1 (Panels A-C) and HSD11B1 (Panels E-G) of the testes from rats treated with 0, 1, and 10 ng/testis MCP-1 from post-EDS day14 to 28 was performed. Panels A and F: the control (0 ng/testis MCP-1); Panels B and G: (1 ng/testis MCP-1); Panels C and H: (10 ng/testis MCP-1; Panels D and H: quantitative data. Black arrow designates CYP11A1 positive Leydig cells. White arrow designatesHSD11B1 positive Leydig cells. Mean ± SEM, *n* = 6, **P < 0.01, ***P < 0.001 when compared to the control.

**Additional file 3: Supplementary Table S1.** Primer information

**Additional file 4: Supplementary Table S2.** Antibodies

**Additional file 5.**

## Data Availability

The datasets used and/or analyzed during the current study are available from the corresponding author on reasonable request.

## Abbreviations

SLC: Stem Leydig Cell
LC: Leydig Cell
EDS: Ethane dimethane sulfonate
T: Testosterone
MCP-1: Monocyte chemoattractant protein-1
CCR2: C-C chemokine receptor type 2
ST: Seminiferous tubule
PDGFRA: Latelet-derived growth factor receptor α
DIM: Differentiation-inducing medium
ITS: Insulin-transferrin-selenium
LH: Luteinizing hormone
LHCGR: Luteinizing hormone receptor
Scarb1: Scavenger receptor class B type I
Star: Steroidogenic acute regulatory protein
CYP11A1: Cytochrome P450 cholesterol side chain cleavage enzyme
3β-HSD1: 3β-hydroxysteroid dehydrogenase1
CYP17A1: Cytochrome P450 17α-hydroxylase
17β-HSD3: 17β-hydroxysteroid dehydrogenase3
11β-HSD1: 11β-Hydroxysteroid Dehydrogenase 1
Rps16: Ribosomal protein S1
Q-PCR: Real-time PCR
BCA: Butyleyanoacrylate
BSA: Albumin from bovine serum
DMSO: Dimethyl sulfoxide
PBS: Phosphate buffer saline
