# Supplementary figures and images for: Monocyte Chemoattractant Protein-1 stimulates the differentiation of rat stem and progenitor Leydig cells during regeneration
Source: BMC Dev Biol. 2020 Oct 6;20:20. doi: 10.1186/s12861-020-00225-1 (PMC7541273; doi:10.1186/s12861-020-00225-1)

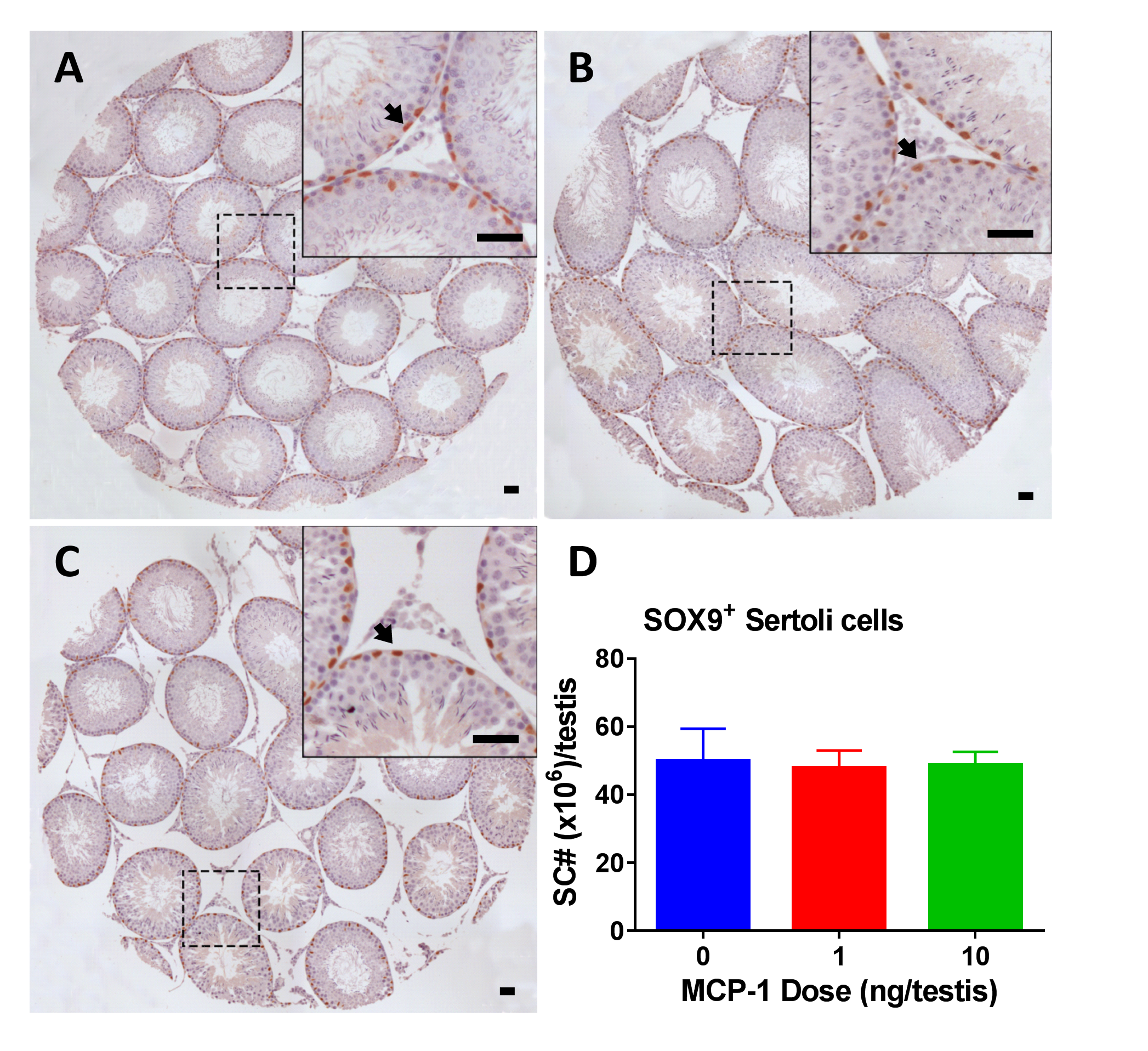

Supplement: Supplementary file 1 — Additional file 1: Supplementary Figure S1. Morphology of Sertoli cells in the testes after in vivo MCP-1 treatment. Immunohistochemical staining of SOX9 (Panels A-C) of the testes from the rats treated with 0, 1, and 10 ng/testis MCP-1 from post-EDS day 14 for 14 days. Black arrow designatesSOX9 positive (brown color in the nucleus) Sertoli cells. Bar = 50 mm. Panel A, the control (0 ng/testis MCP-1); Panel B, 1 ng/ testis MCP-1; Panel C, 10 ng/testis MCP-1; Panel D, quantitative data. Mean ± SEM, n = 4–6. No significant difference was observed. [file 12861_2020_225_MOESM1_ESM.tif]

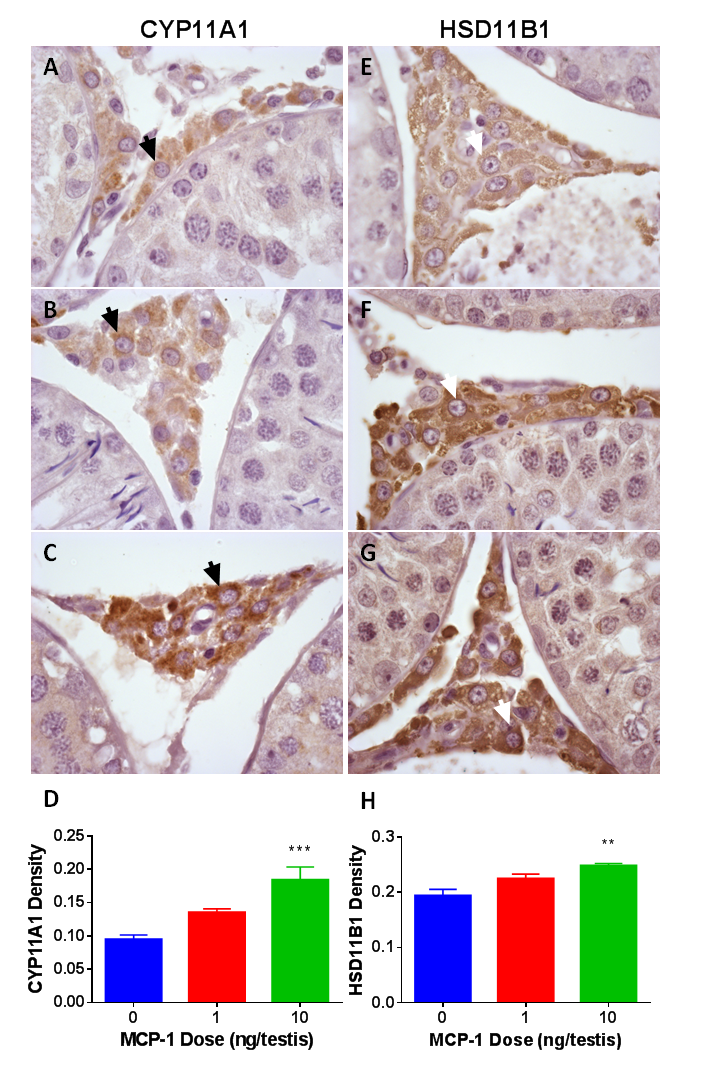

Supplement: Supplementary file 2 — Additional file 2: Supplementary Figure S2. Semi-quantitative measurement of CYP11A1 and HSD11B1 levels of the Leydig cells after in vivo MCP-1 treatment. Immunohistochemical staining of CYP11A1 (Panels A-C) and HSD11B1 (Panels E-G) of the testes from rats treated with 0, 1, and 10 ng/testis MCP-1 from post-EDS day14 to 28 was performed. Panels A and F: the control (0 ng/testis MCP-1); Panels B and G: (1 ng/testis MCP-1); Panels C and H: (10 ng/testis MCP-1; Panels D and H: quantitative data. Black arrow designates CYP11A1 positive Leydig cells. White arrow designatesHSD11B1 positive Leydig cells. Mean ± SEM, n = 6, **P < 0.01, ***P < 0.001 when compared to the control. [file 12861_2020_225_MOESM2_ESM.tif]
