# Supplementary material for: Monocyte Chemoattractant Protein-1 stimulates the differentiation of rat stem and progenitor Leydig cells during regeneration
Source: BMC Dev Biol. 2020 Oct 6;20:20. doi: 10.1186/s12861-020-00225-1 (PMC7541273; doi:10.1186/s12861-020-00225-1)
Supplement: Supplementary file 3 — Additional file 3: Supplementary Table S1. Primer information [file 12861_2020_225_MOESM3_ESM.doc]

**Supplementary Table S1**. Primer information

| **Primer**  **Symbol** | **Gene name** | **Primer direction** | **Sequences (5’to 3’)** | **PCR**  **(bp)** | **Accession** |
| --- | --- | --- | --- | --- | --- |
| Lhcgr | Luteinizing hormone receptor | Forward | CTGCGCTGTCCTGGCC | 103 | NM_012978 |
|  |  | Reverse | CGACCTCATTAAGTCCCCTGAA |  |  |
| Scarb1 | Scavenger receptor class B, member 1 | Forward | ATGGTACTGCCGGGCAGAT | 117 | NM_031541 |
|  |  | Reverse | CGAACACCCTTGATTCCTGGTA |  |  |
| Star | Steroidogenic acute regulatory protein | Forward | CCCAAATGTCAAGGAAATCA | 187 | NM_031558 |
|  |  | Reverse | AGGCATCTCCCCAAAGTG |  |  |
| Cyp11a1 | Cholesterol side chain cleavage enzyme | Forward | AAGTATCCGTGATGTGGG | 127 | NM_017286 |
|  |  | Reverse | TCATACAGTGTCGCCTTTTCT |  |  |
| Hsd3b1 | 3β-Hydroxysteroid dehydrogenase 1 | Forward | CCCTGCTCTACTGGCTTGC | 189 | NM_001007719 |
|  |  | Reverse | TCTGCTTGGCTTCCTCCC |  |  |
| Cyp17a1 | 17α-hydroxylase/ 17,20-lyase | Forward | TGGCTTTCCTGGTGCACAATC | 90 | NM_012753 |
|  |  | Reverse | TGAAAGTTGGTGTTCGGCTGAAG |  |  |
| Hsd17b3 | 17β-Hydroxysteroid dehydrogenase 3 | Forward | TGAAAGTTGGTGTTCGGCTGAAG | 202 | NM_054007 |
|  |  | Reverse | TGAAAGTTGGTGTTCGGCTGAAG |  |  |
| Hsd11b1 | [11β-Hydroxysteroid dehydrogenase 1](https://www.baidu.com/link?url=RASn5FVJOHQO5F8yLZuLK2GaE-txBvaDG-Aix0zS1TOK-H6BDM3SQ-dtmZqJCiib&wd=&eqid=adb6e42d00037867000000045854b8a0) | Forward | GAAGAAGCATGGAGGTCA | 290 | NM_017080 |
|  |  | Reverse | CTCAAGATTATCCCAGAGG |  |  |
| Insl3 | Insulin-like 3 | Forward | GTGGCTGGAGCAACGACA | 102 | NM_053680 |
|  |  | Reverse | AGAAGCCTGGTGAGGAAGC |  |  |
| Nr5a1 | Nuclear receptor  steroidogenic factor 1 | Forward  Reverse | CAGAGCTGCAAAATCGACAA  CCCGAATCTGTGCTTTCTTC | 186 | NM_053344 |
| Rps16 | Ribosomal protein  S16 | Forward | AAGTCTTCGGACGCAAGAAA | 148 | [NM_001169146](https://www.ncbi.nlm.nih.gov/entrez/viewer.fcgi?db=nucleotide&id=310703681) |
|  |  | Reverse | TTGCCCAGAAGCAGAACAG |  |  |
