# Supplementary material for: Monocyte Chemoattractant Protein-1 stimulates the differentiation of rat stem and progenitor Leydig cells during regeneration
Source: BMC Dev Biol. 2020 Oct 6;20:20. doi: 10.1186/s12861-020-00225-1 (PMC7541273; doi:10.1186/s12861-020-00225-1)
Supplement: Supplementary file 4 — Additional file 4: Supplementary Table S2. Antibodies [file 12861_2020_225_MOESM4_ESM.doc]

**Supplementary Table S2. Antibodies**

| **Antibody** | **Species** | **Vendor (City, State, catalogue)** | **Dilution** | |
| --- | --- | --- | --- | --- |
|  |  |  | **WB** | **HS** |
| ACTB | rabbit | Cell Signaling Technology (Danvers, MA) | 1:1000 | ND |
| LHCGR | rabbit | Multi Sciences (Hangzhou, China) | 1:1000 | ND |
| SCARB1 | rabbit | Multi Sciences (Hangzhou, China) | 1:1000 | ND |
| CYP11A1 | rabbit | Cell Signaling Technology (Danvers, MA) | 1:1000 | 1:500 |
| HSD3B1 | rabbit | Abcam (San Francisco, CA) | 1:2000 | ND |
| CYP17A1 | rabbit | Abcam (San Francisco, CA) | 1:1000 | ND |
| CYP17B3 | rabbit | Abcam (San Francisco, CA) | 1:1000 | ND |
| HSD11B1 | rabbit | Abcam (San Francisco, CA) | ND | 1: 500 |
| pERK1/2 | mouse | Abcam (San Francisco, CA) | 1:10000 | ND |
| ERK1/2 | mouse | Abcam (San Francisco, CA) | 1:1000 | ND |
| PCNA | mouse | Abcam (San Francisco, CA) | ND | 1:50 |
| SMA | mouse | Sigma Aldrich(Saint Louis, A2547) | ND | 1:200 |

ND = Not detected; WB = Western blot; HS = Histochemical staining.
