# Supplementary material for: Monocyte Chemoattractant Protein-1 stimulates the differentiation of rat stem and progenitor Leydig cells during regeneration
Source: BMC Dev Biol. 2020 Oct 6;20:20. doi: 10.1186/s12861-020-00225-1 (PMC7541273; doi:10.1186/s12861-020-00225-1)

FIG4. ACTB invivo-rs

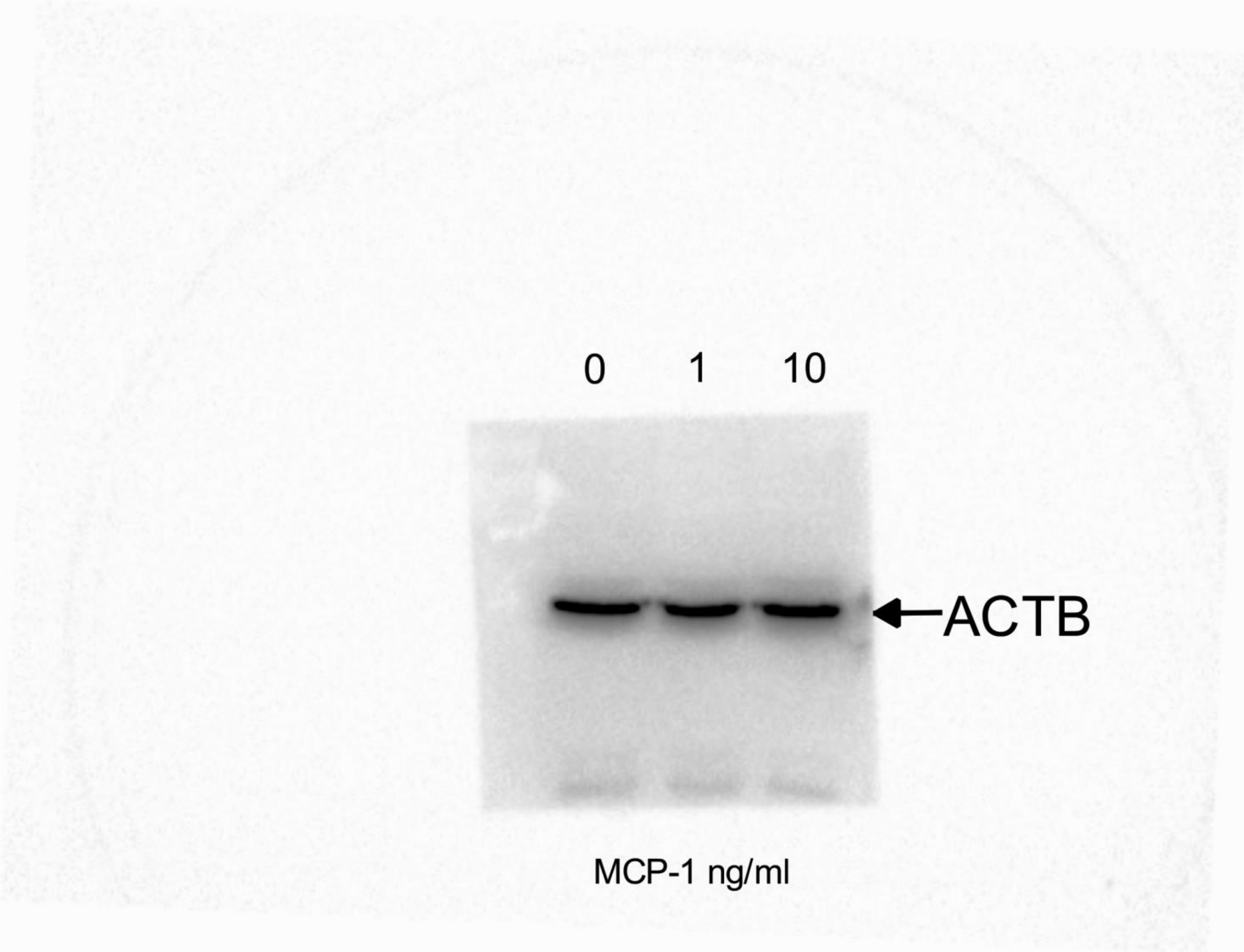

FIG4. HSD11B1 invivo-rs Exposure_1.0sec

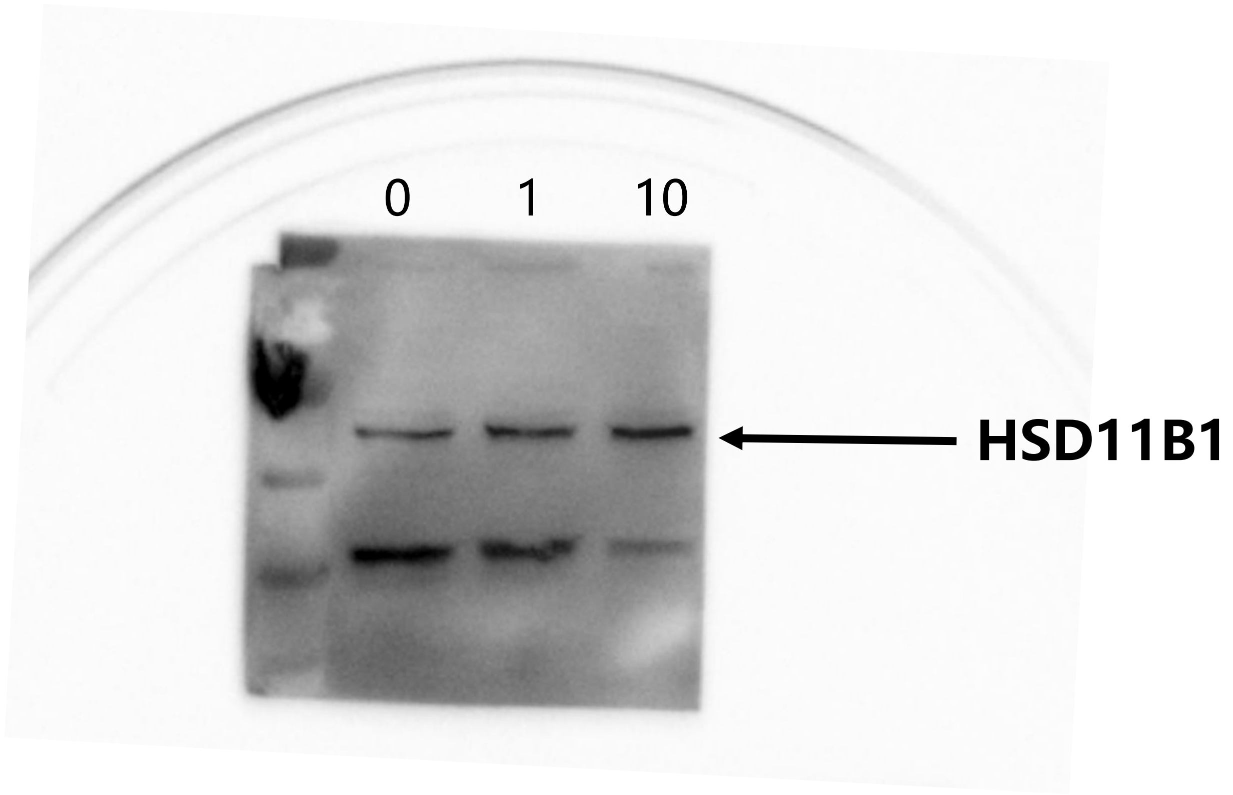

MCP-1 ng/ml

FIG4. HSD11B1 invivo-rs Exposure_1.5sec

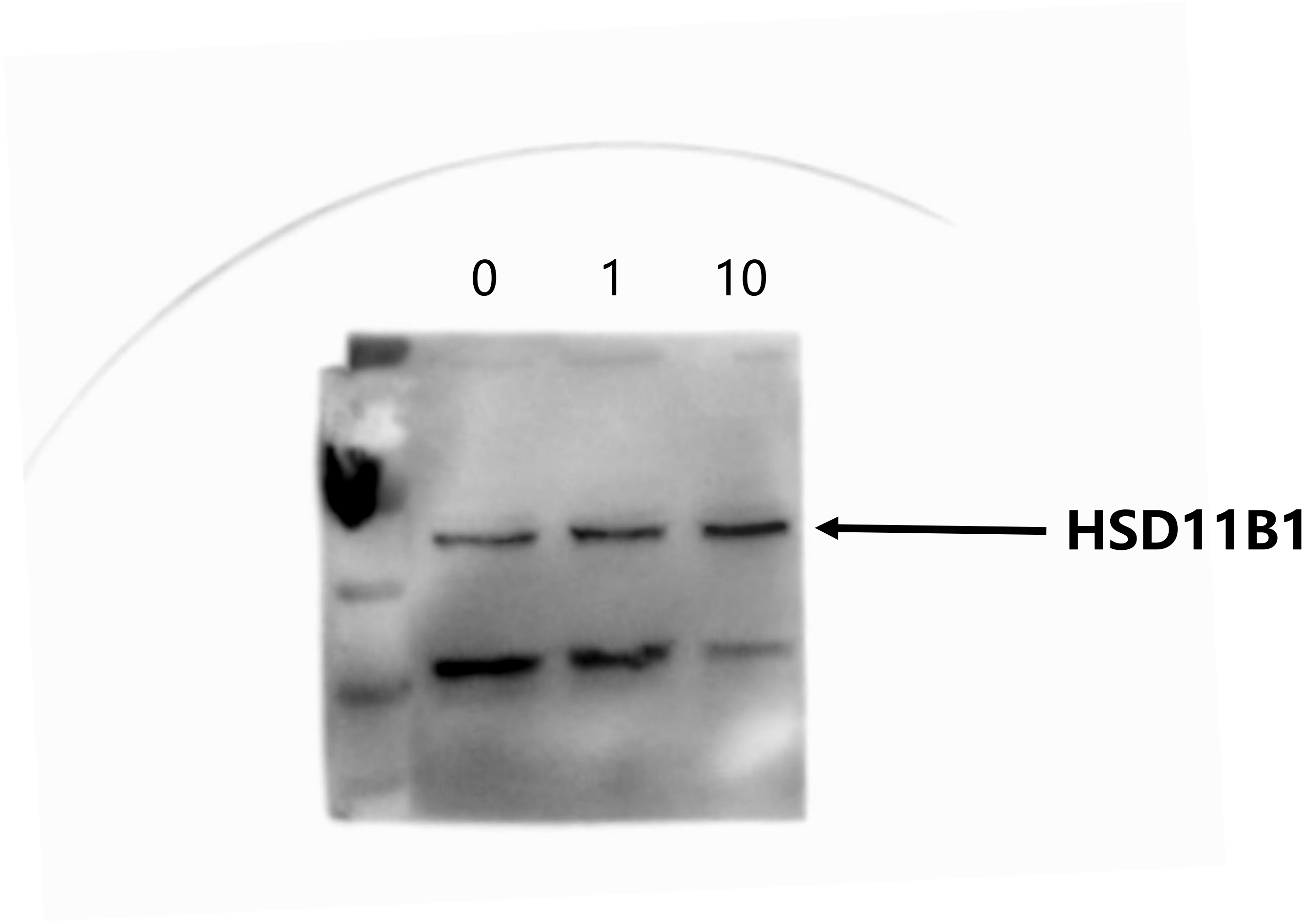

MCP-1 ng/ml

FIG4. CYP11A1 invivo-rs

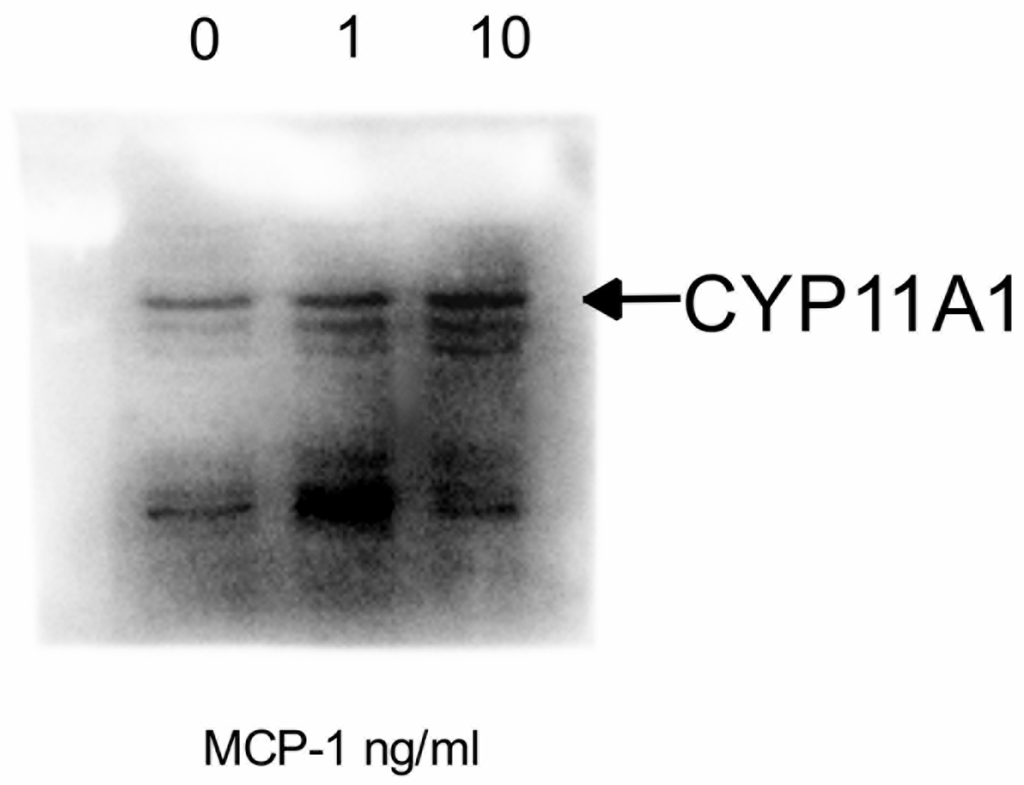

FIG4. CYP17A1 invivo-rs Exposure_6.0sec

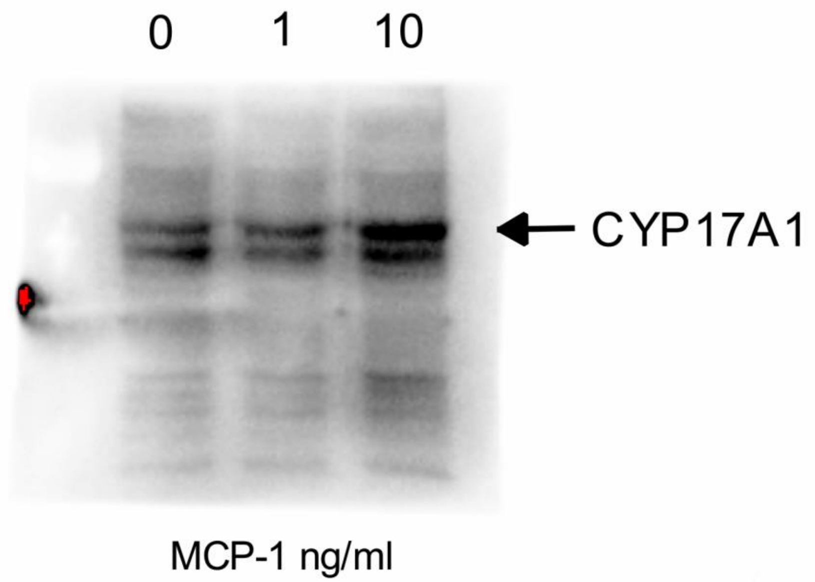

FIG4. CYP17A1 invivo-rs Exposure_5.0sec

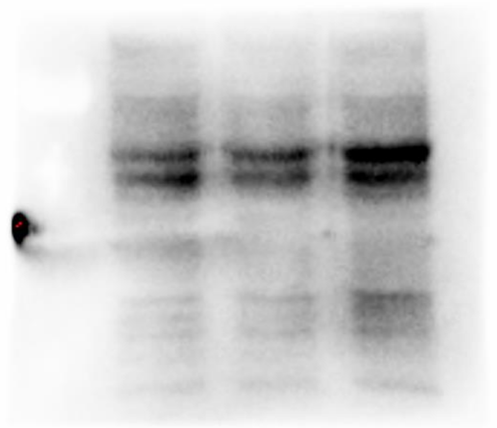

FIG4. CYP17A1 invivo-rs Exposure_4.0sec

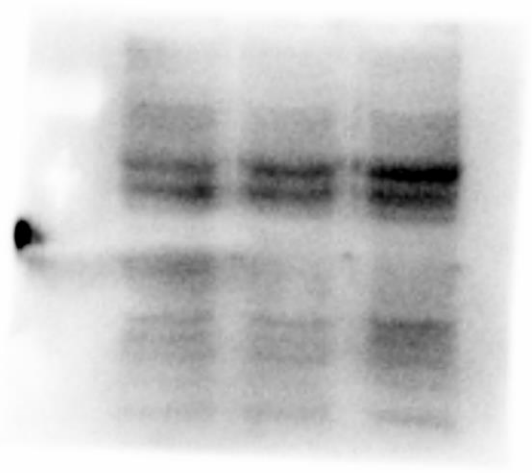

FIG4. HSD3B1 invivo Exposure_15.0sec

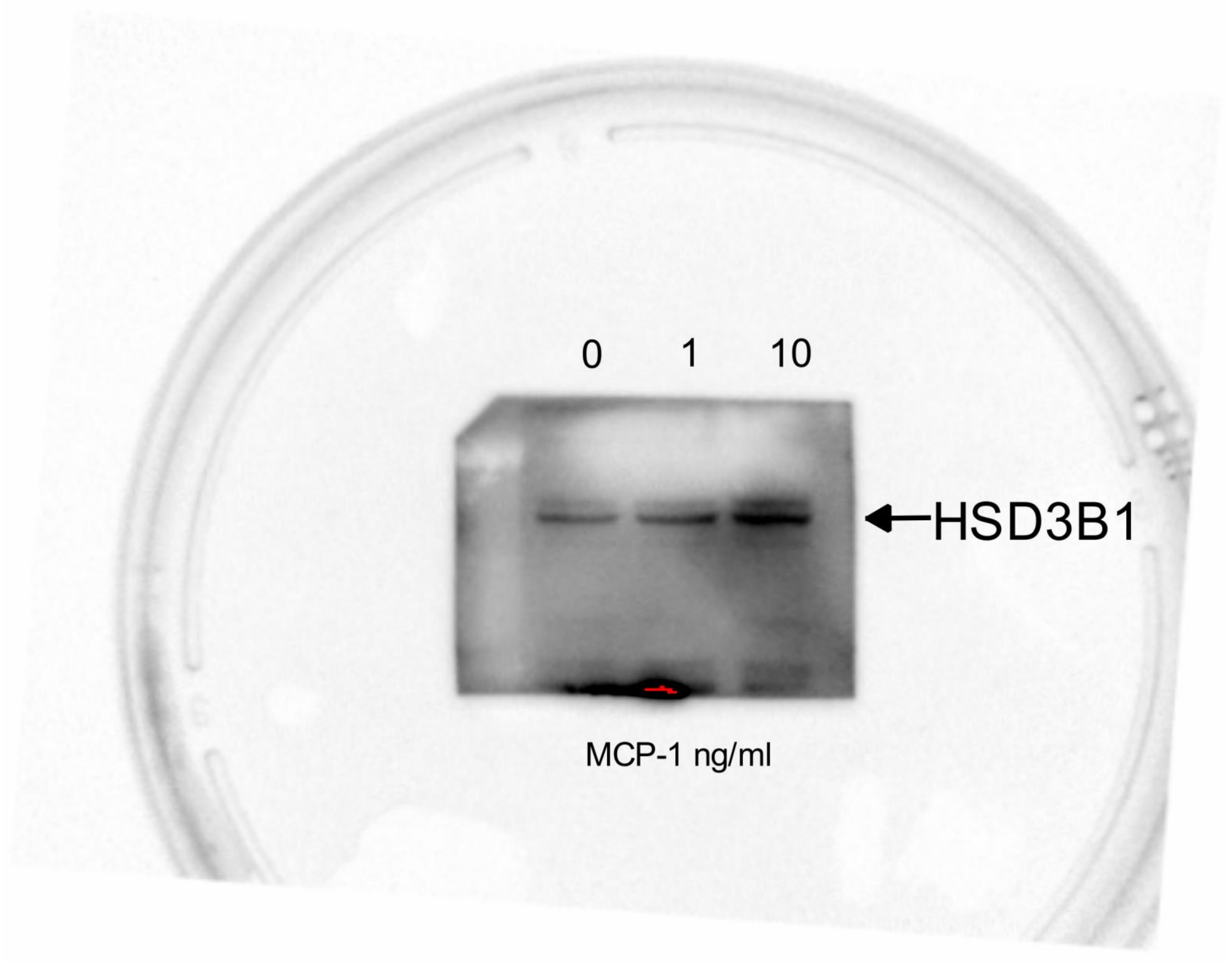

FIG4. HSD3B1 invivo Exposure_11.7sec

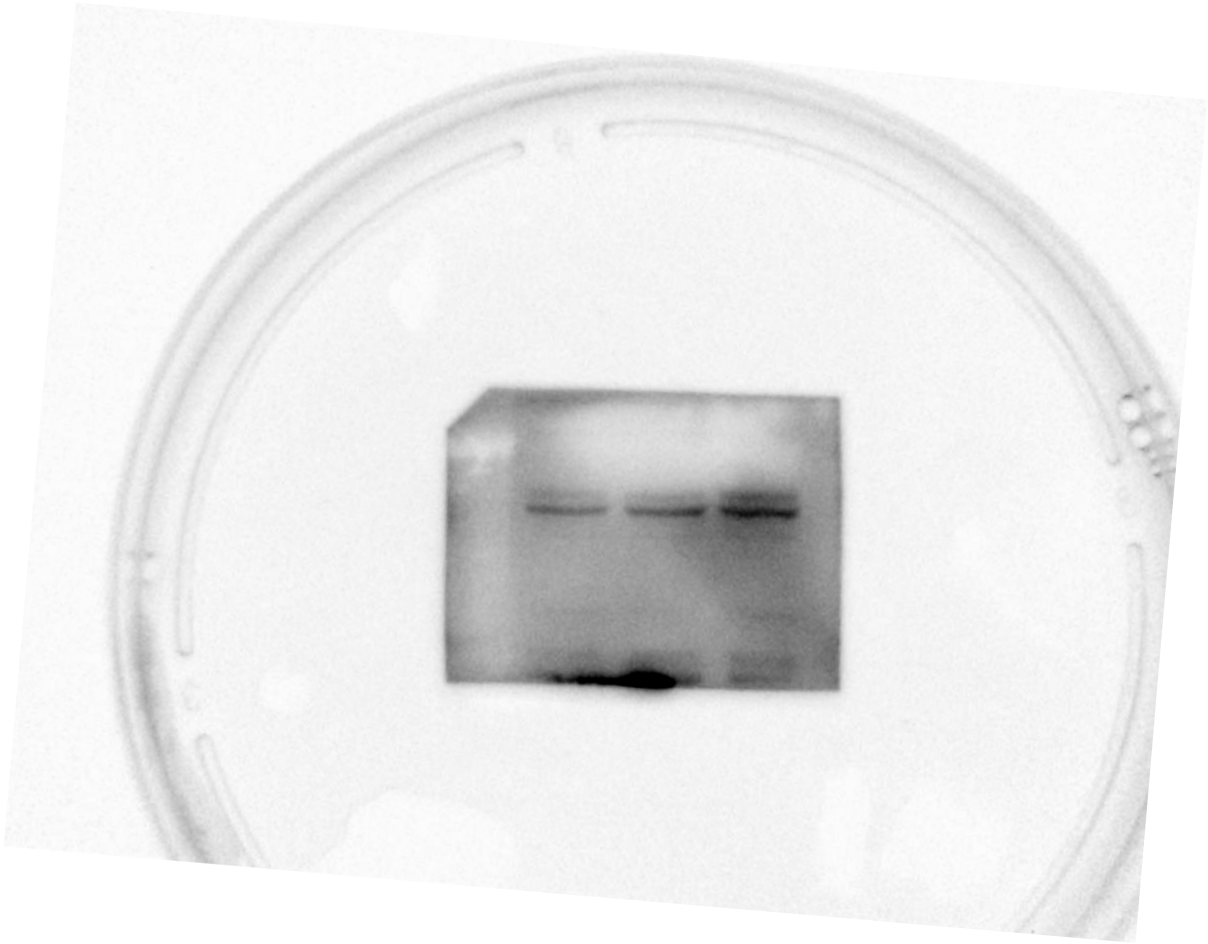

FIG4. HSD3B1 invivo Exposure_10.0sec

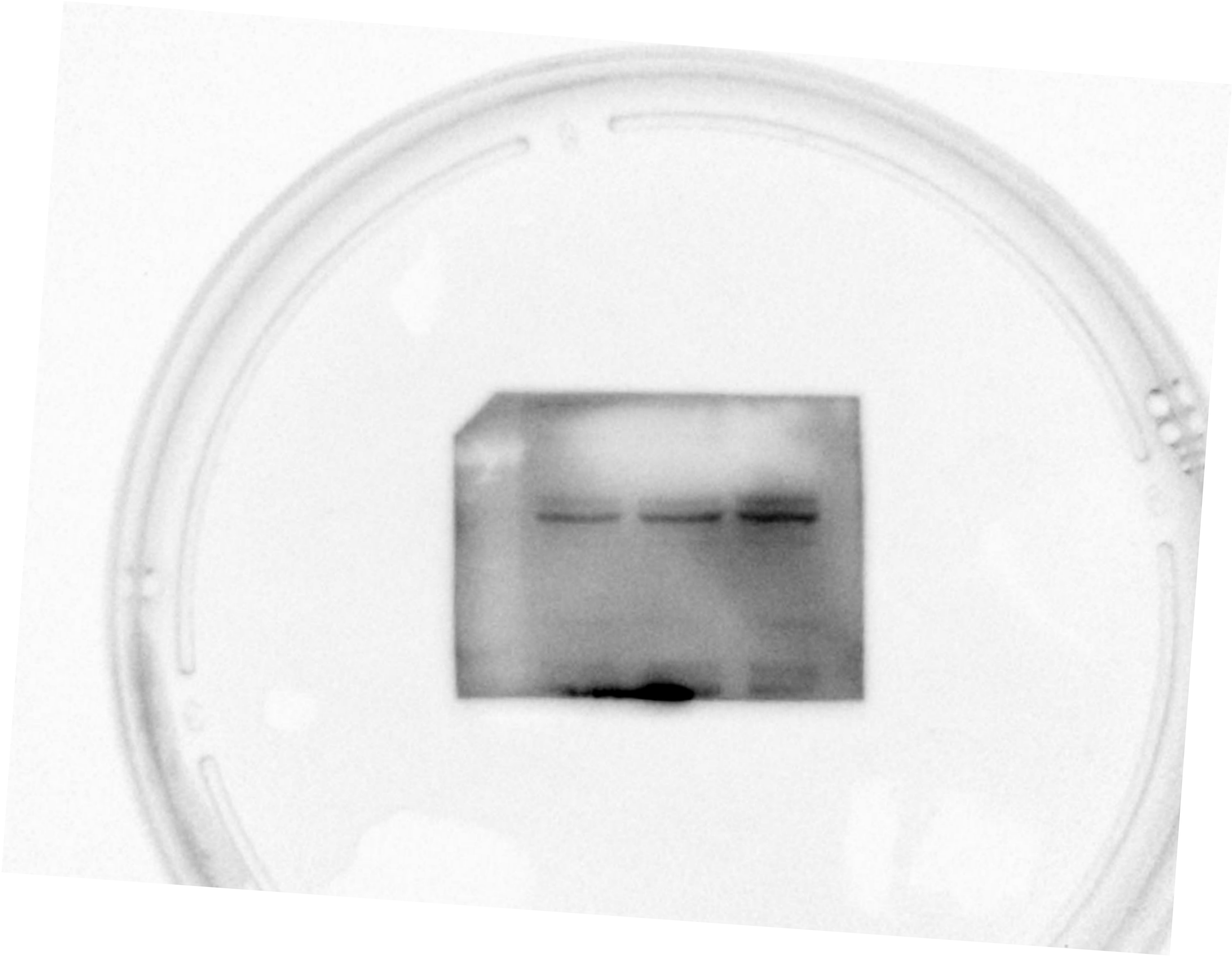

FIG4. HSD17B3 invivo Exposure_35.0sec

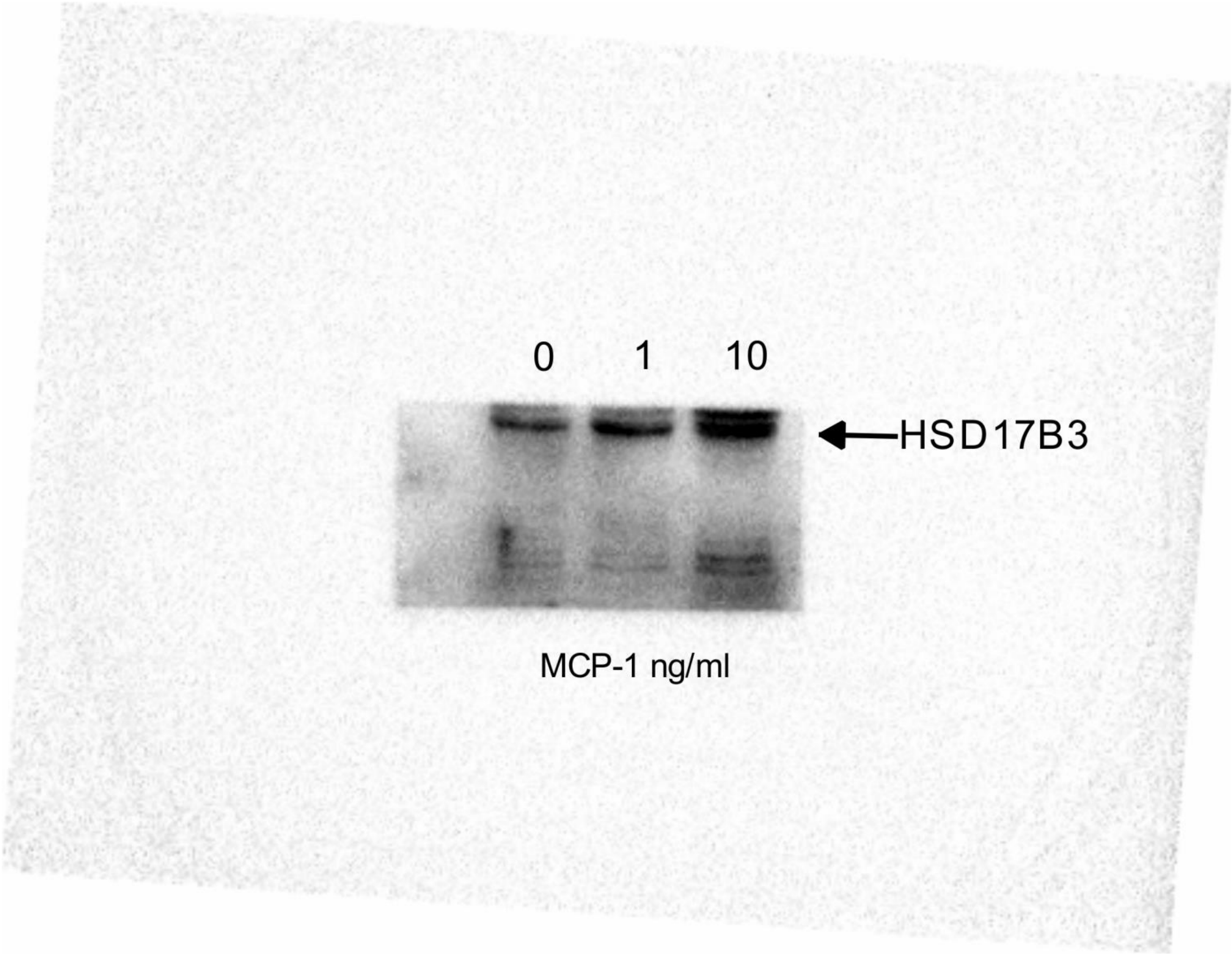

FIG4. HSD17B3 invivo Exposure_22.1sec

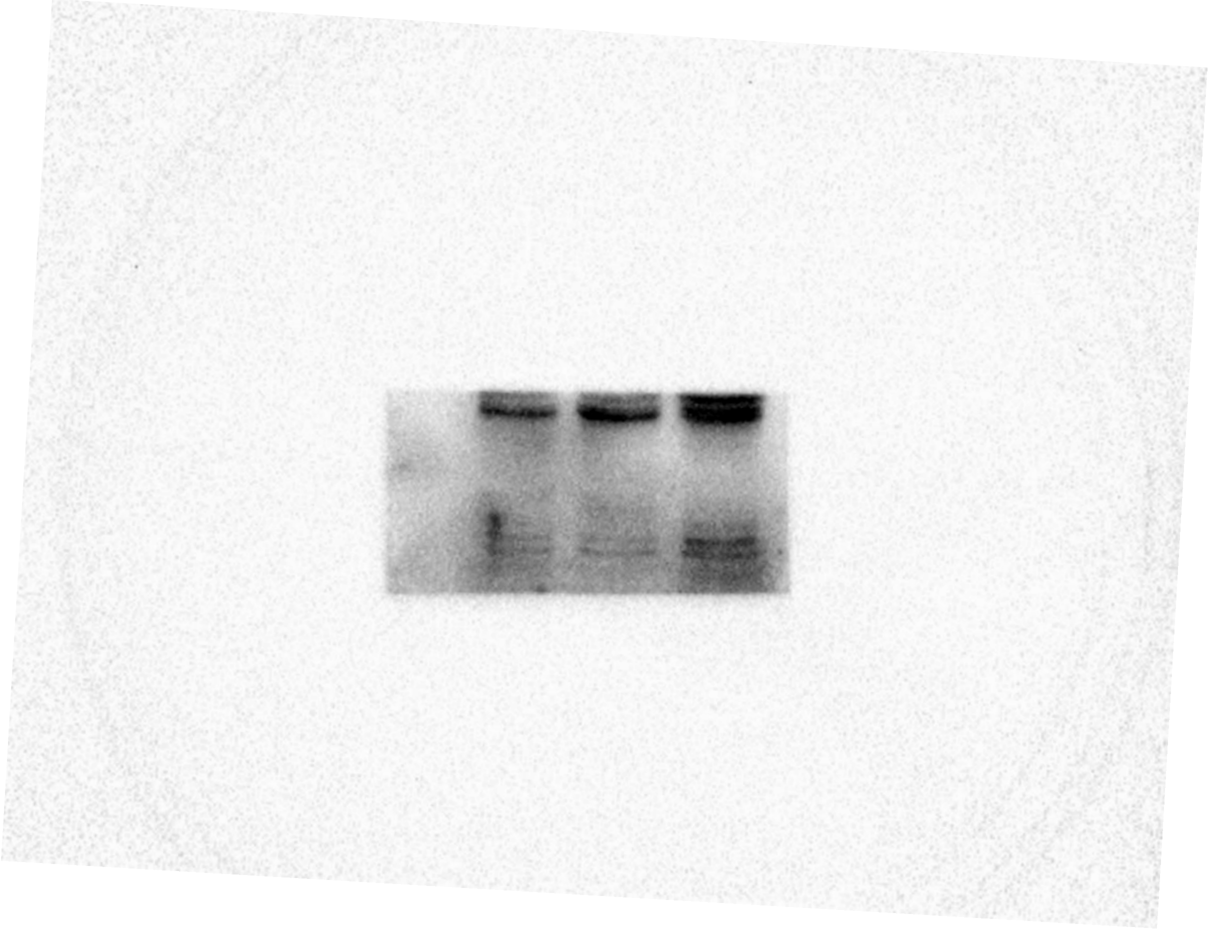

FIG4. HSD17B3 invivo Exposure_20.0sec

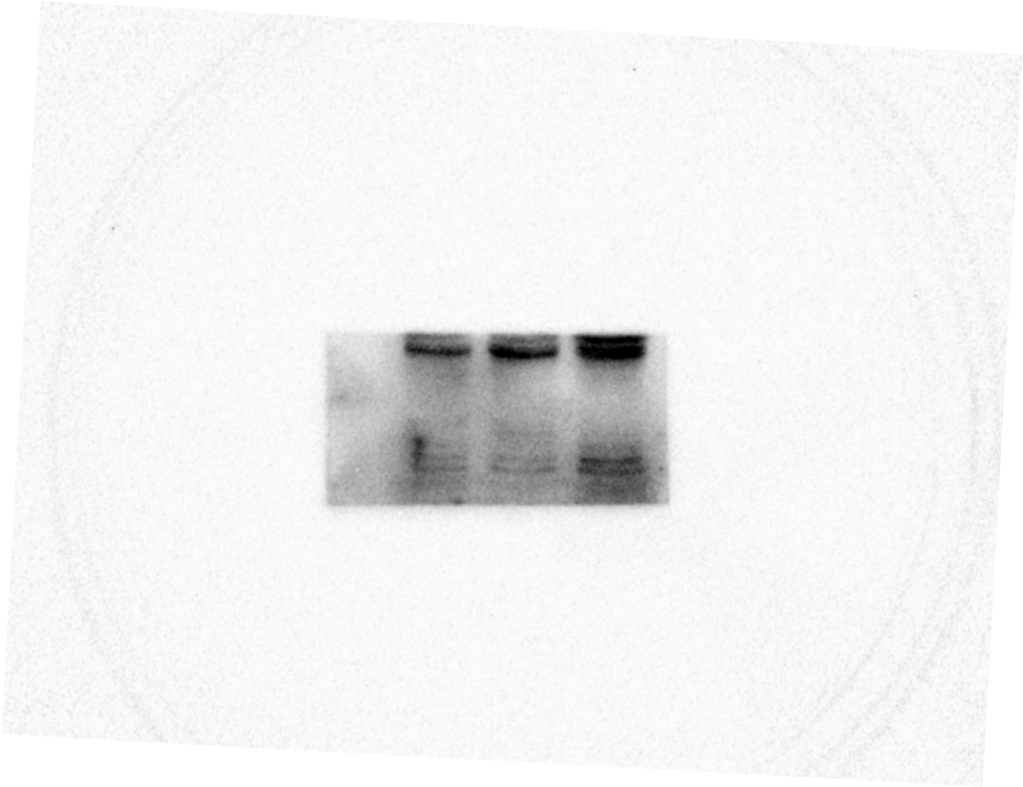

FIG4. LHCGR invivo Exposure_4.5sec

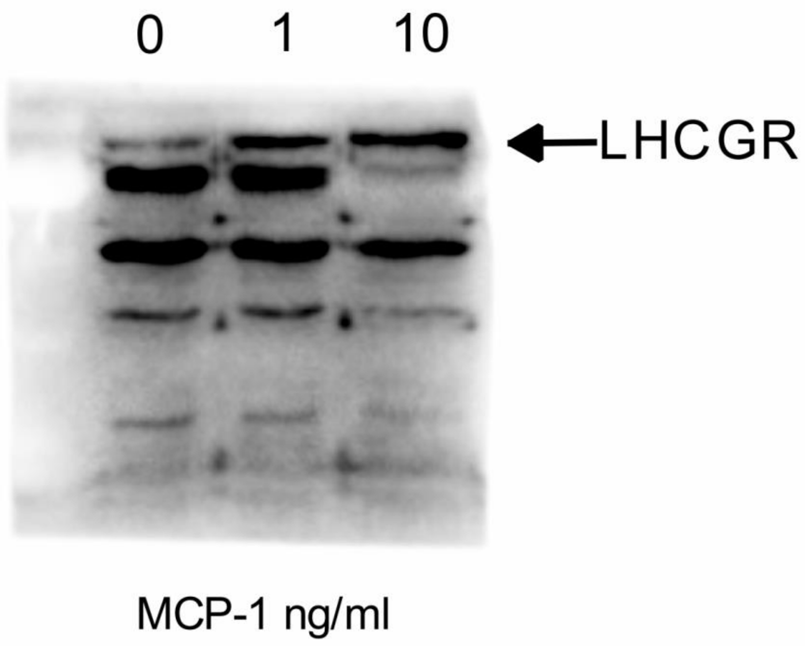

FIG4. LHCGR invivo Exposure_3.2sec

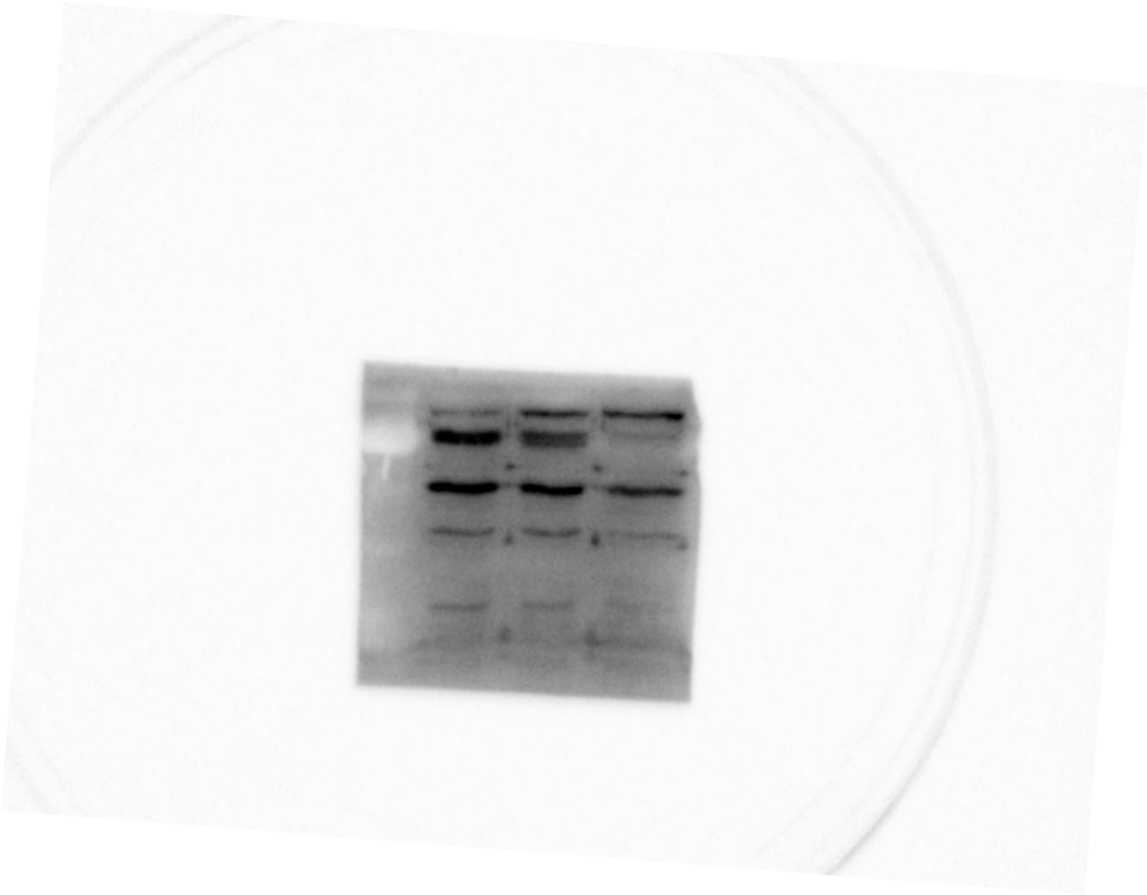

FIG4. LHCGR invivo Exposure_2.0sec

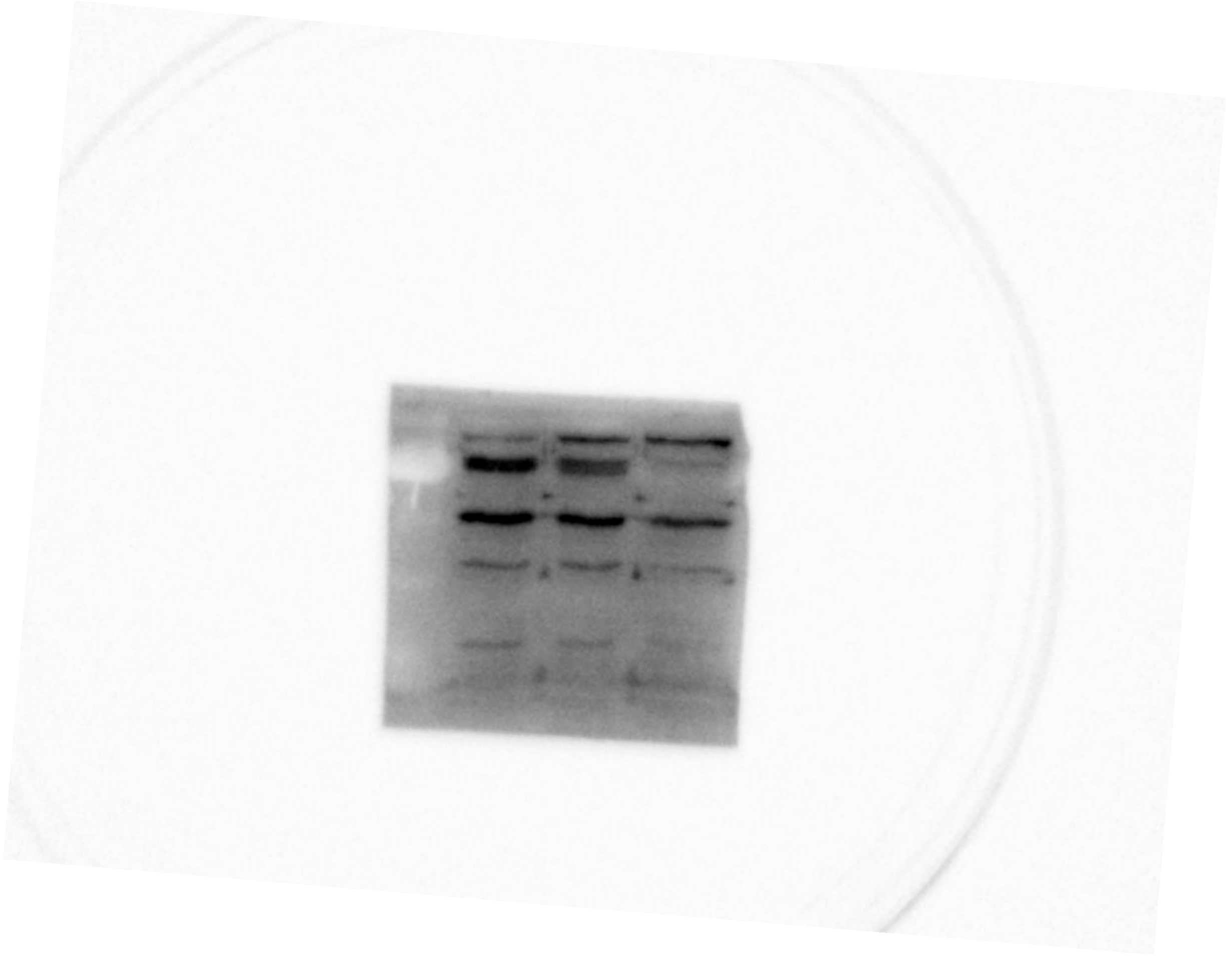

FIG4. SCARB1 invivo-rs Exposure_1.0sec

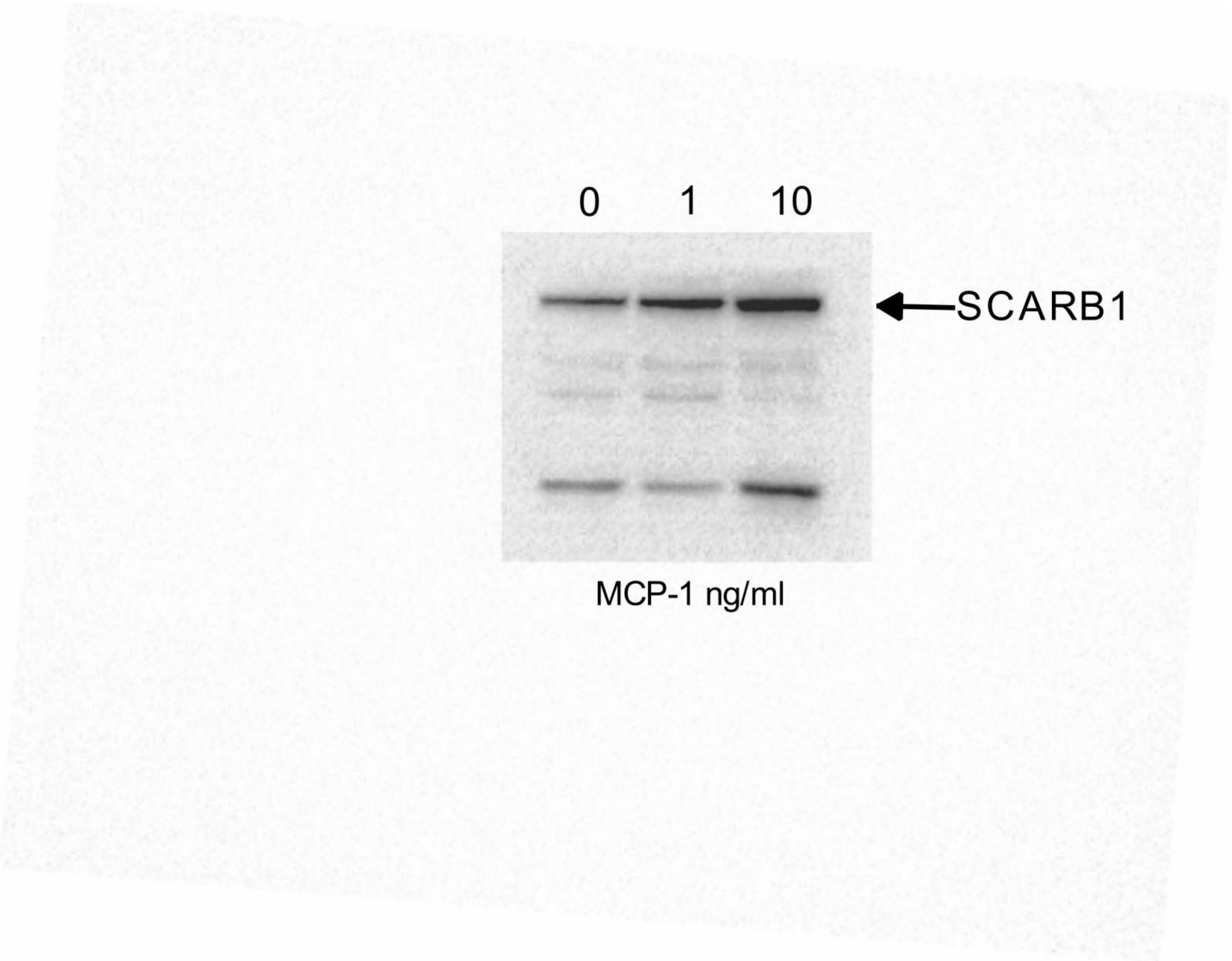

FIG4. SCARB1 invivo-rs Exposure_2.0sec

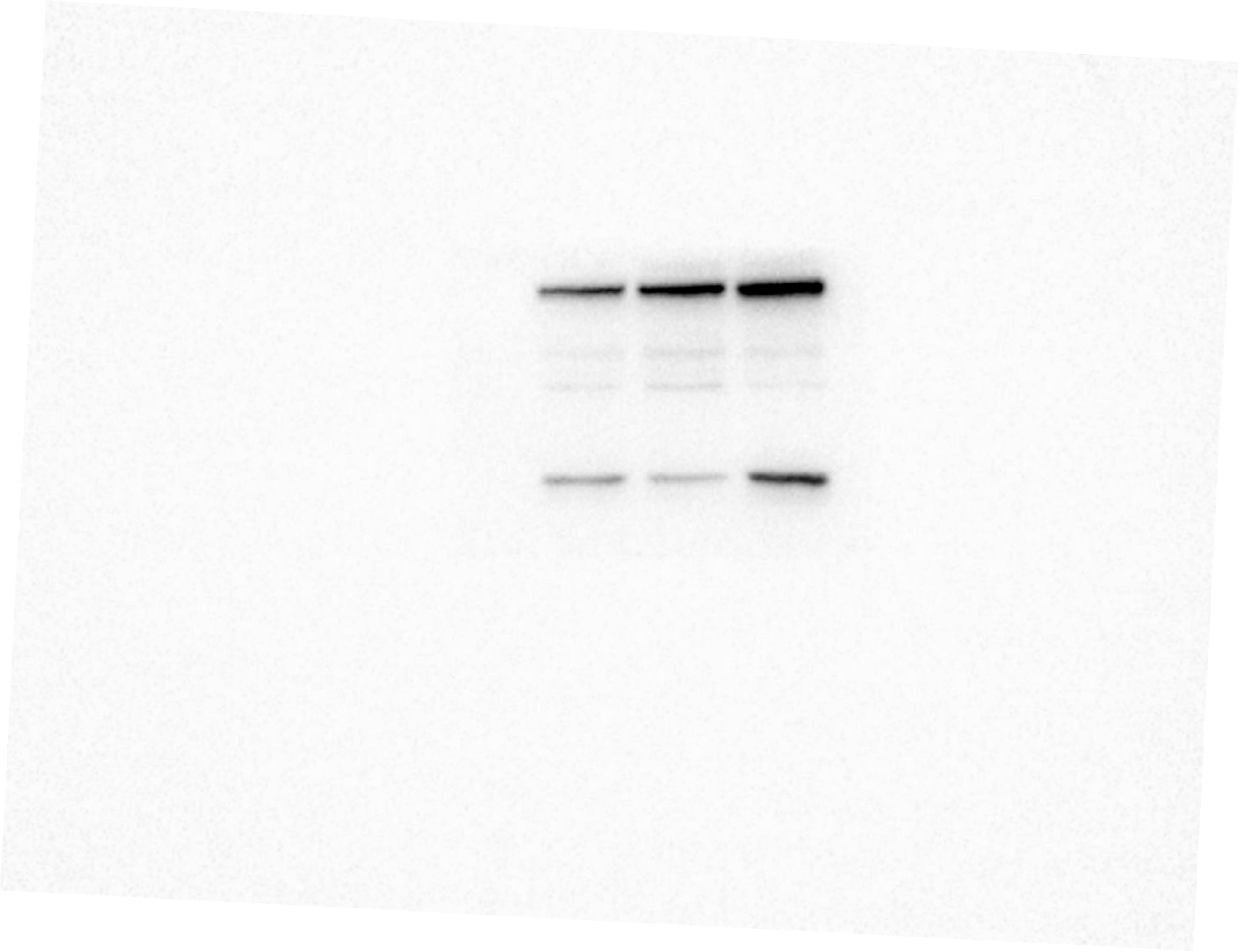

FIG4. SCARB1 invivo-rs Exposure_5.0sec

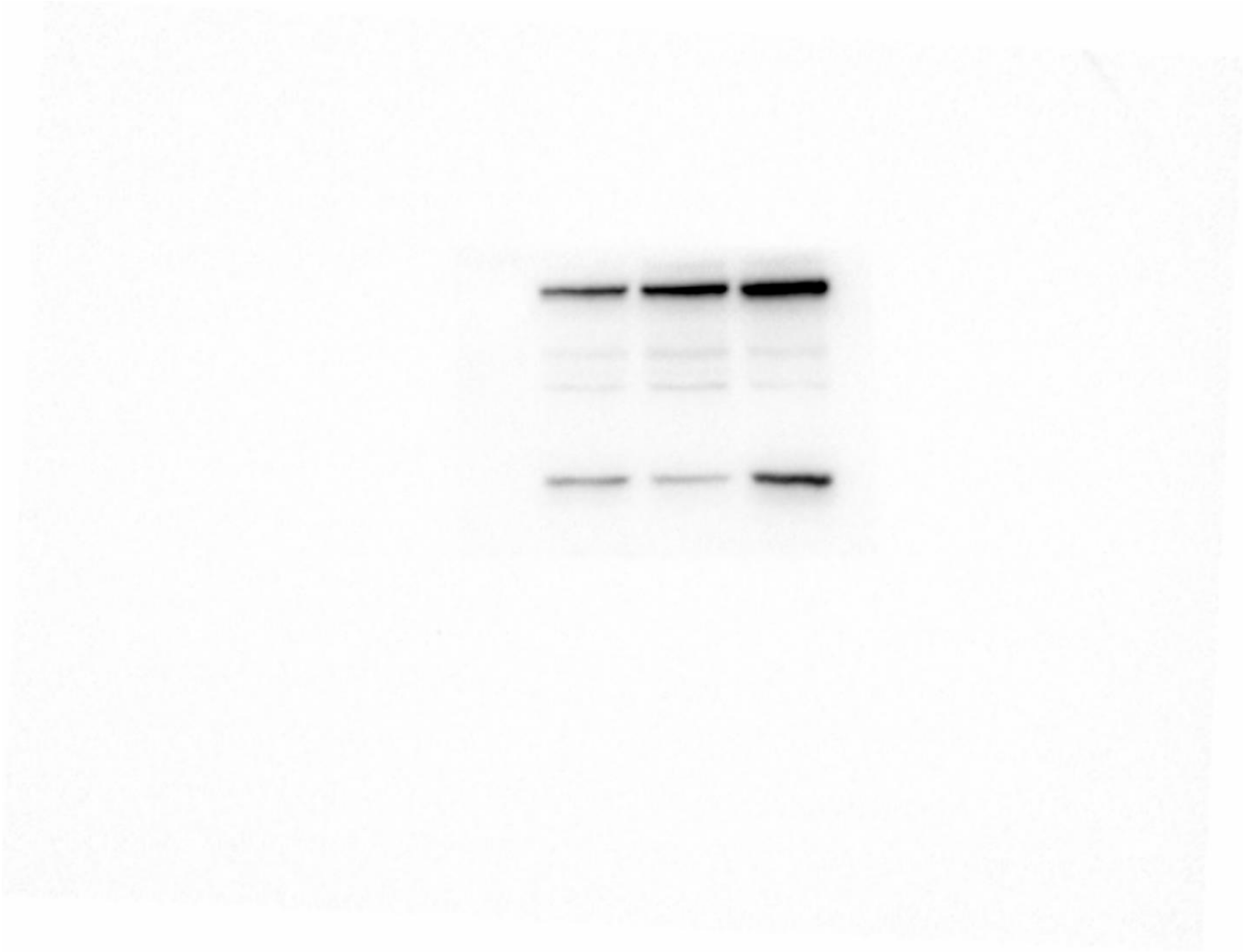

FIG5. ACTB invivo Exposure_17sec

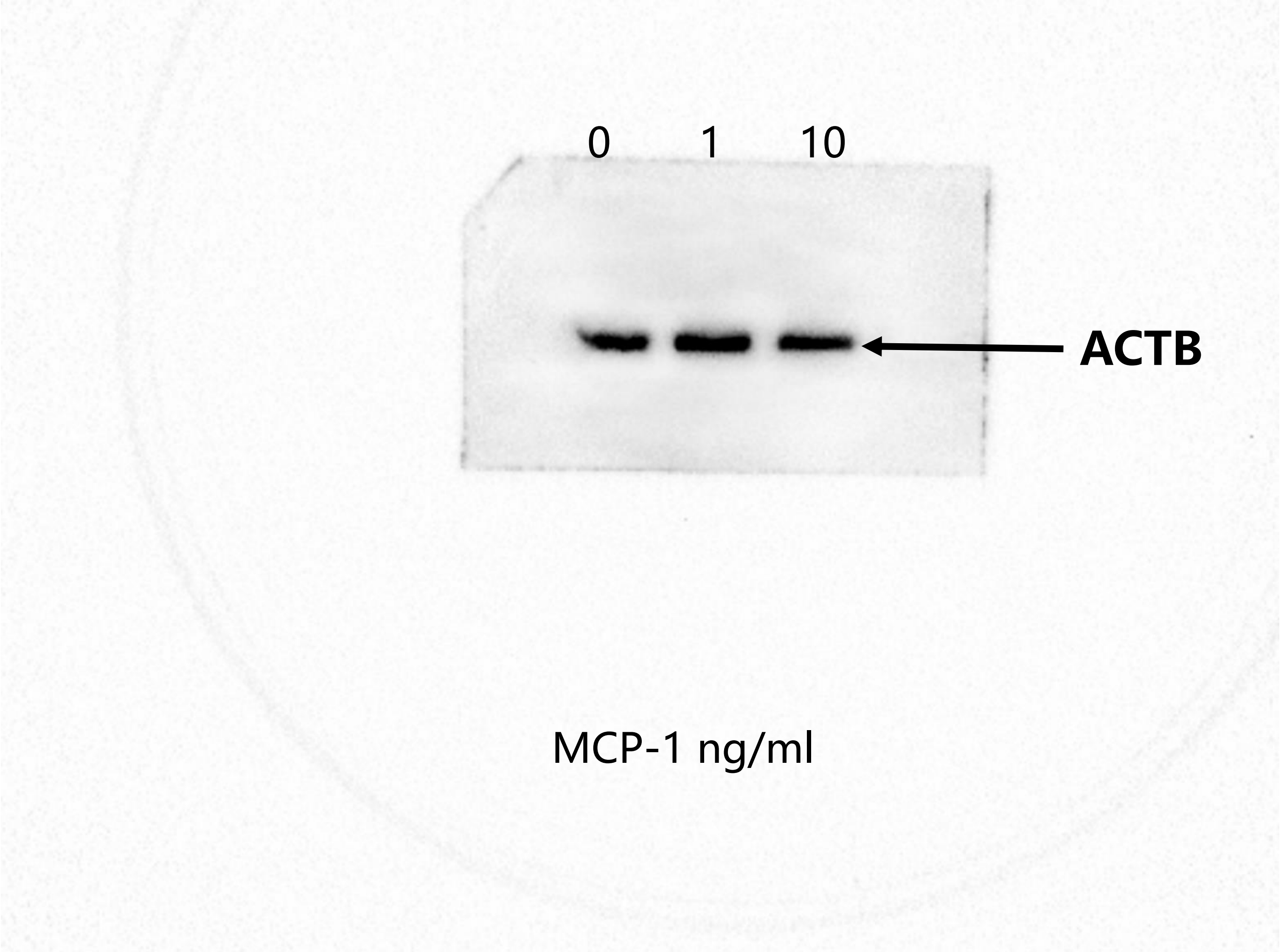

FIG5. ERK invivo Exposure_25sec

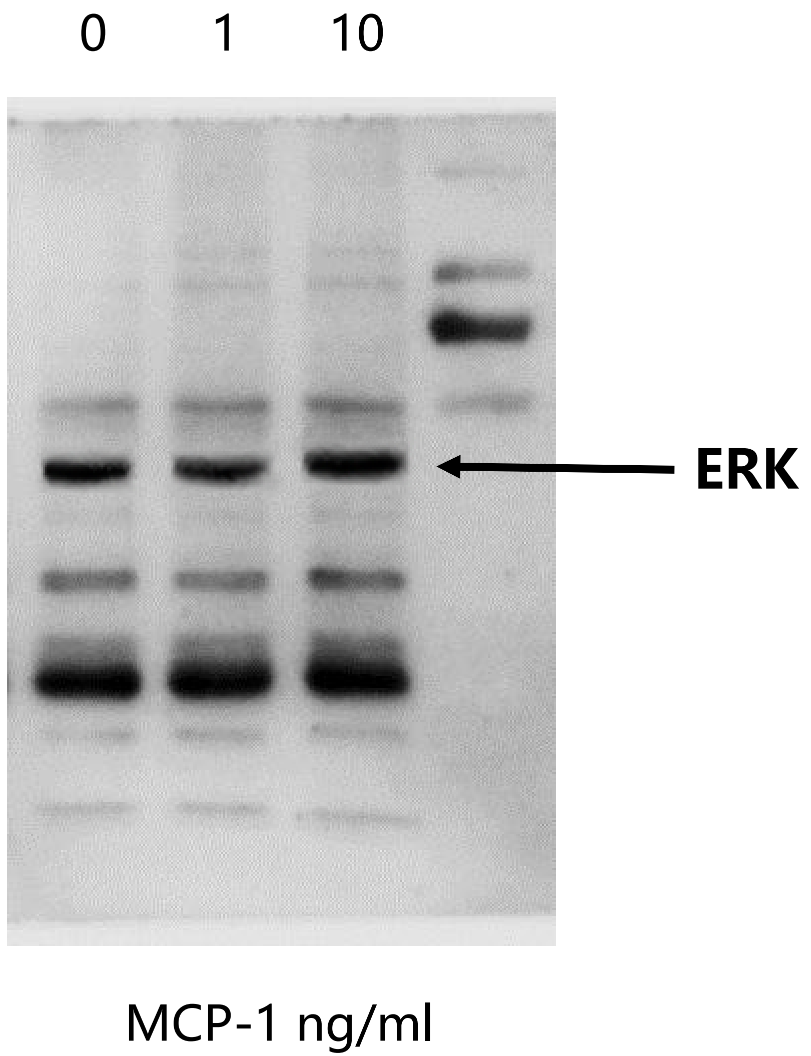

FIG5. pERK invivo Exposure_30sec

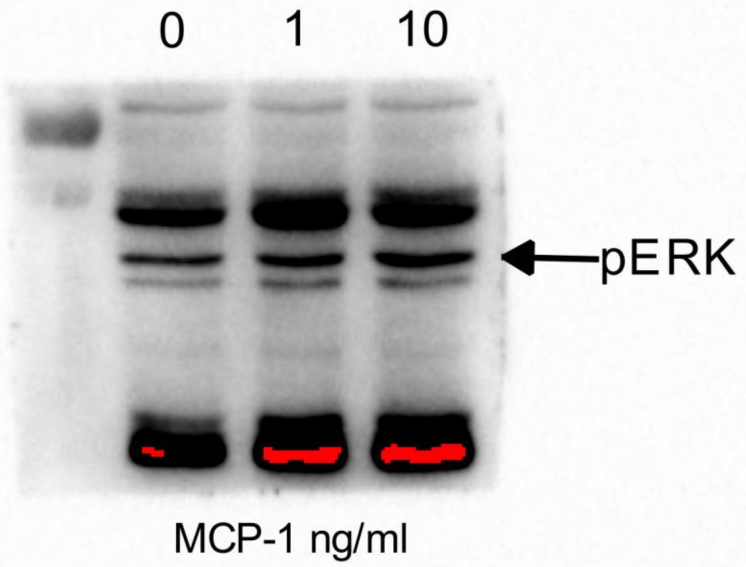

FIG5. pERK invivo Exposure_25sec

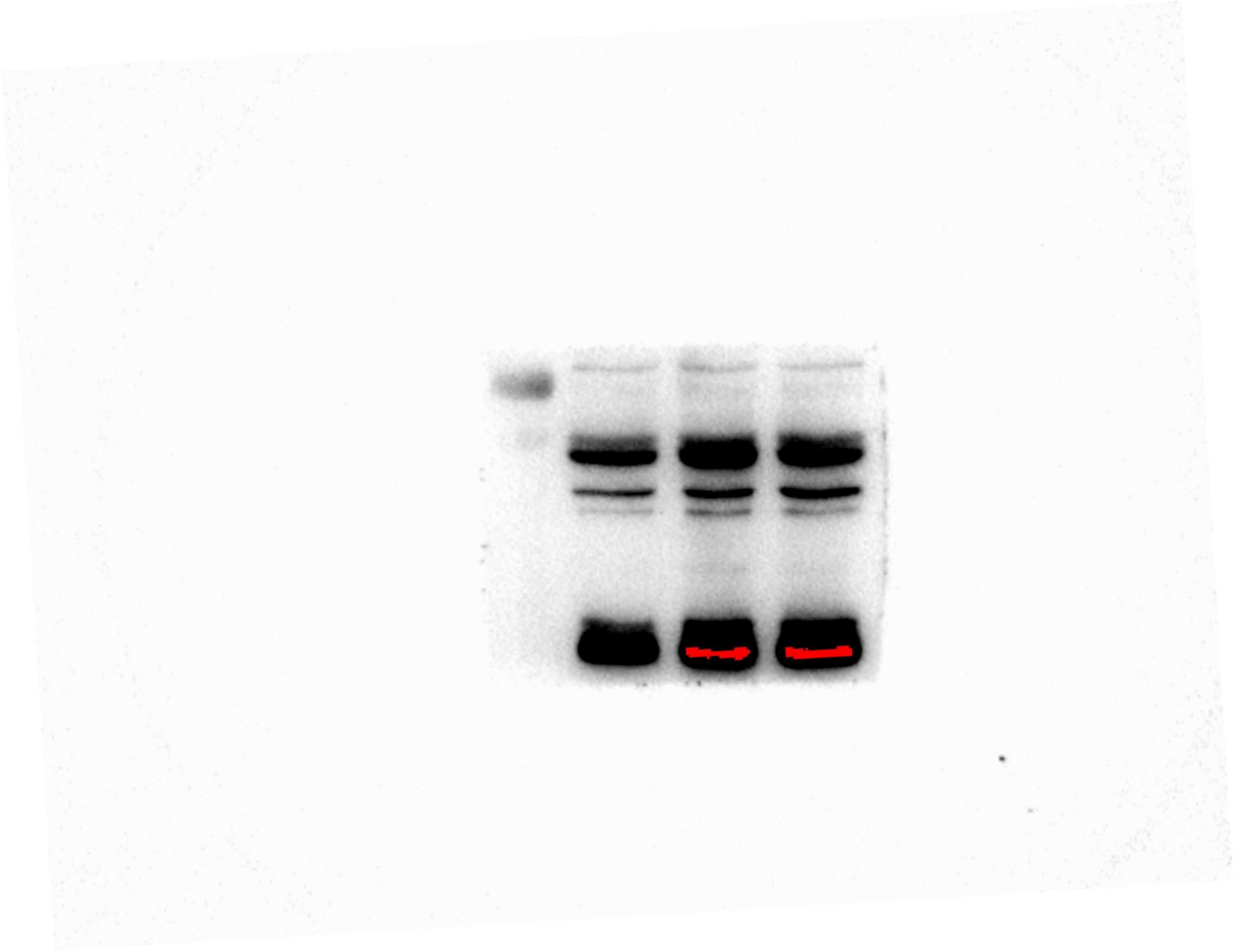

FIG5. pERK invivo Exposure_13sec

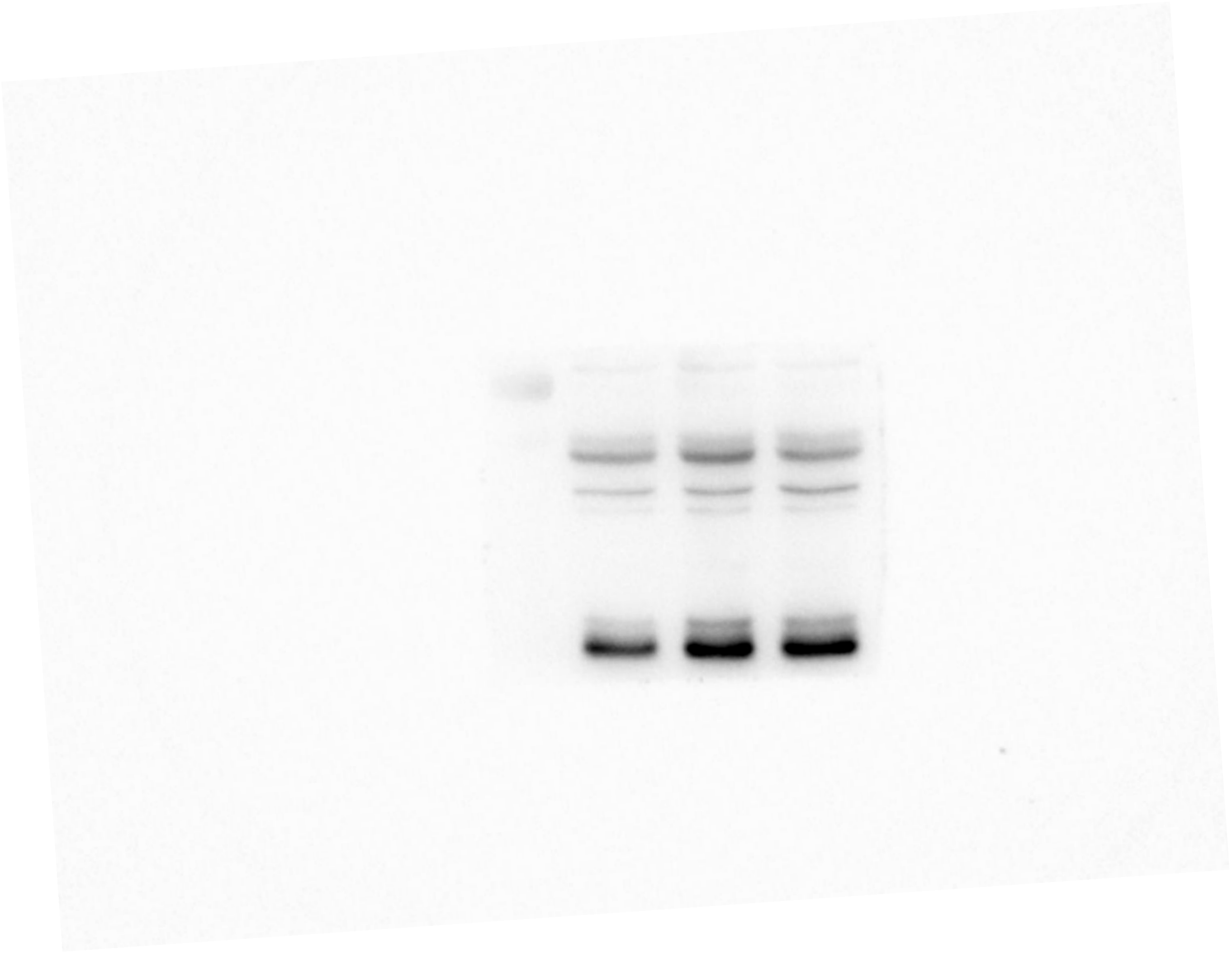

FIG9.ACTB invtro

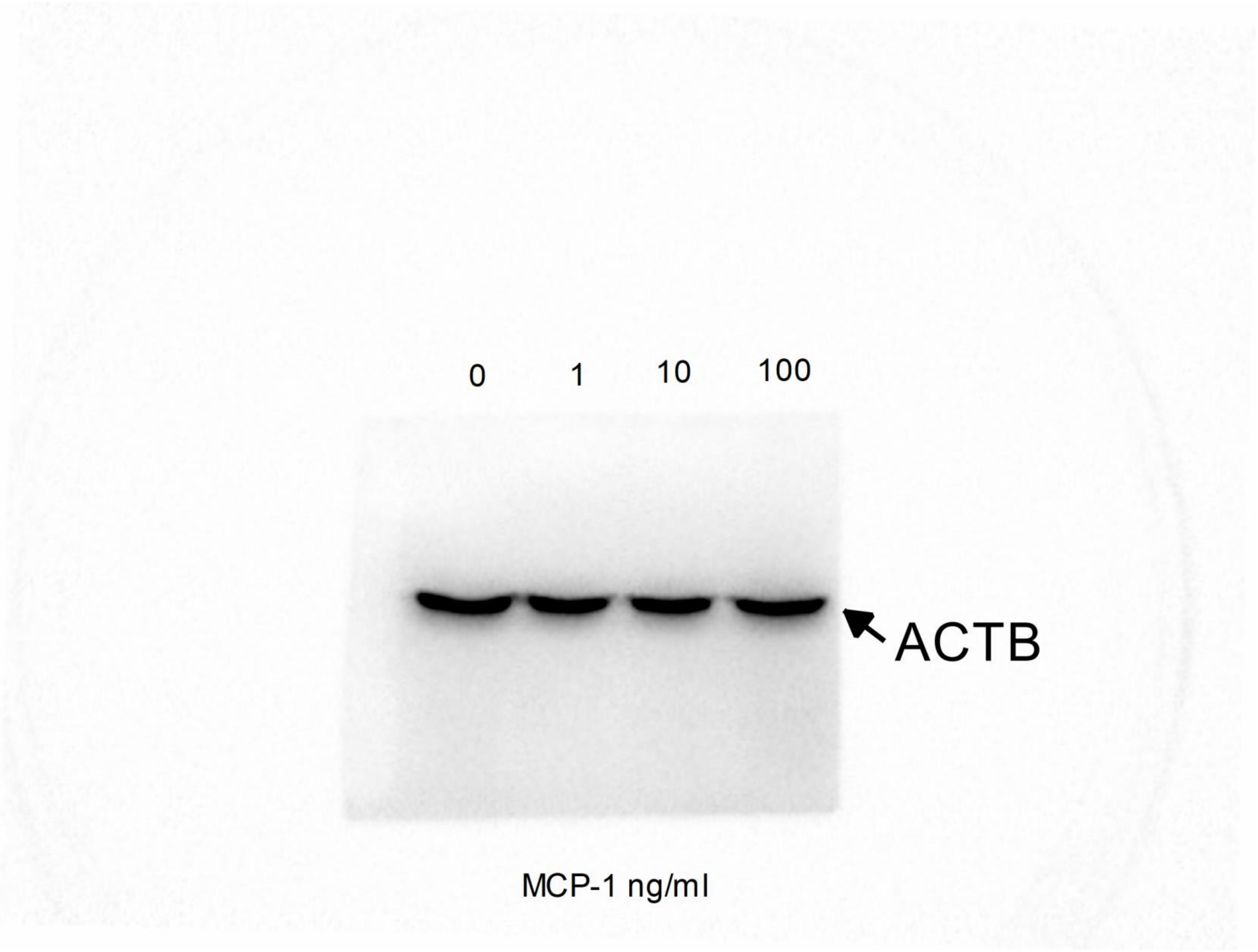

FIG9.CYP11A1 invtro

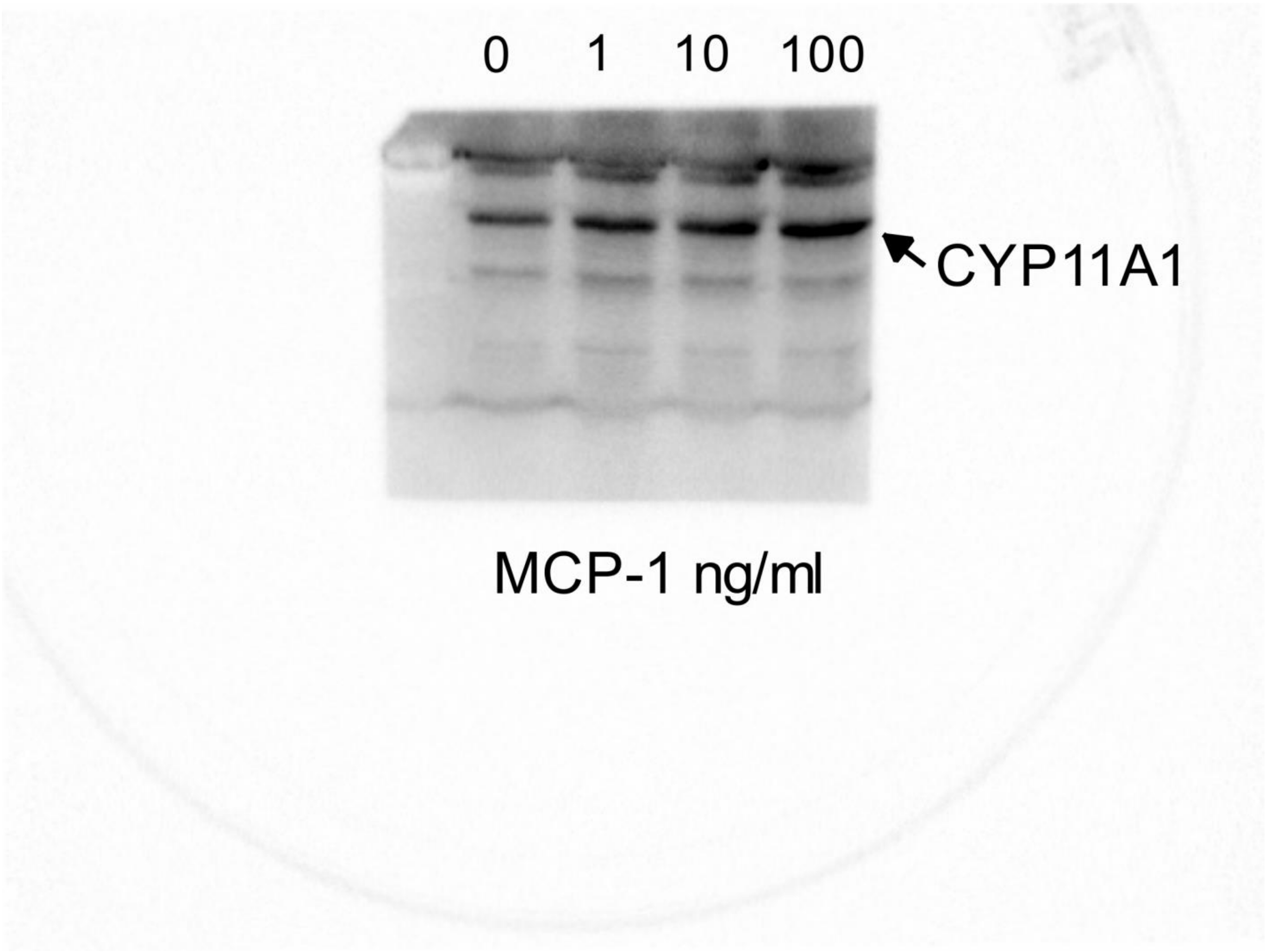

FIG9.HSD-3β invtro-rs

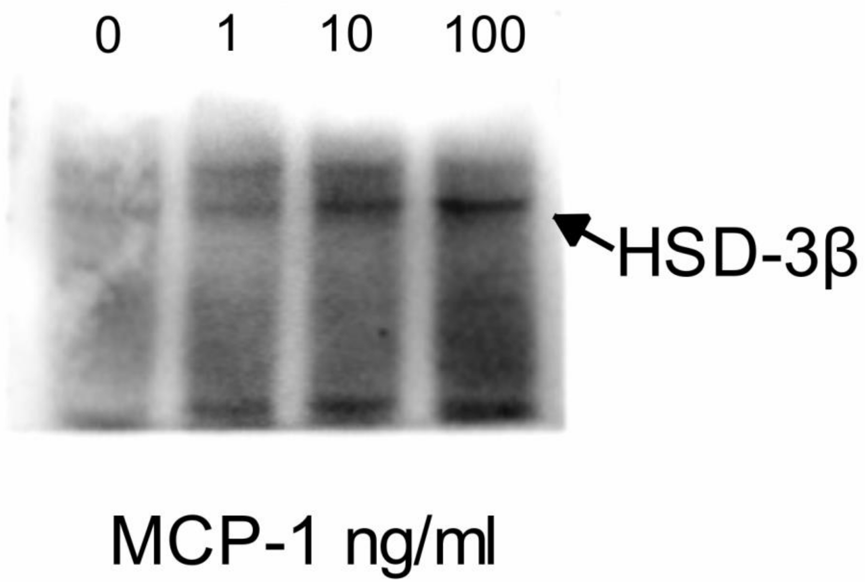

FIG9.LHCGR invtro

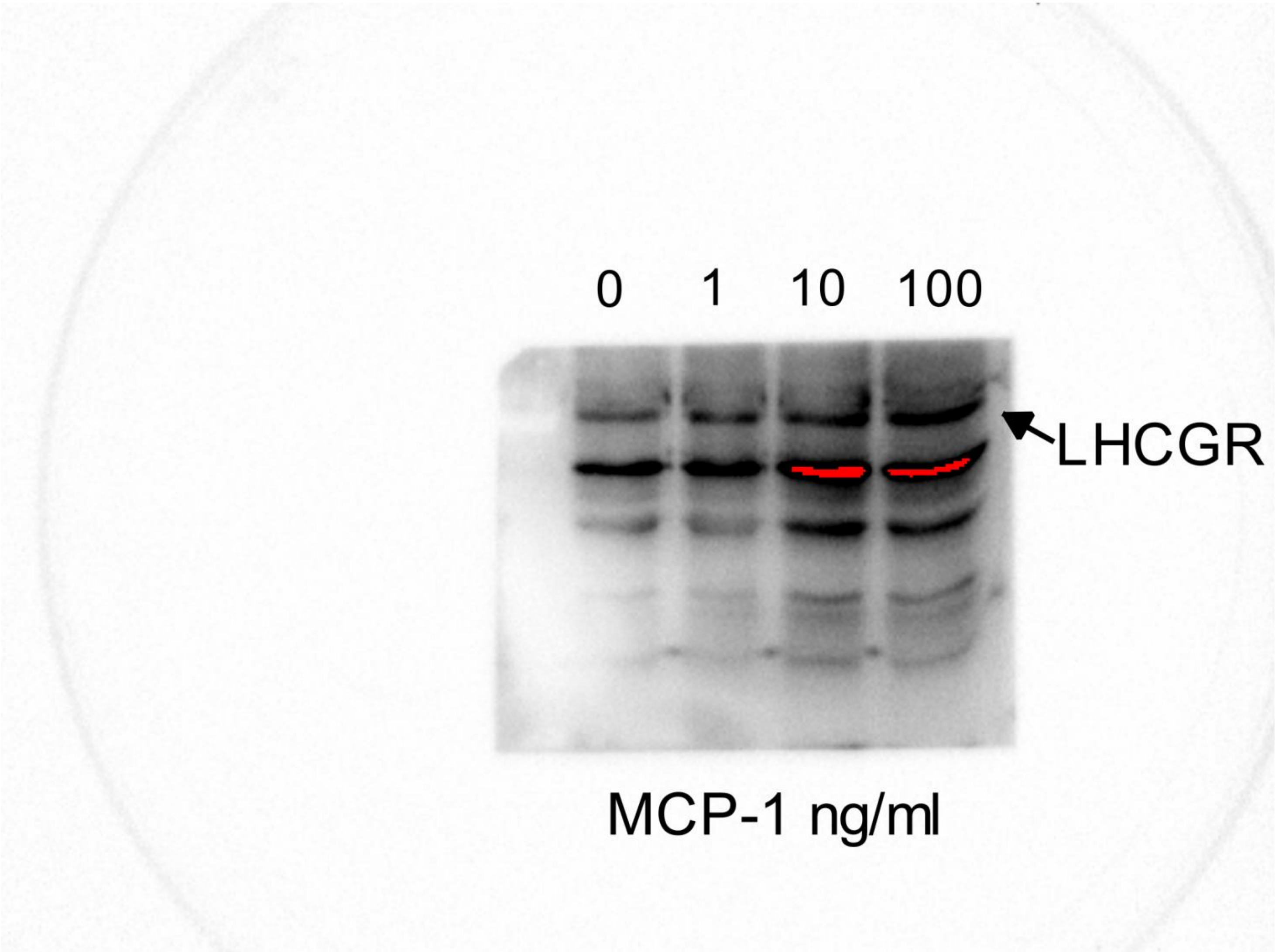

Supplement: Supplementary file 5 — Additional file 5. [file 12861_2020_225_MOESM5_ESM.pdf]
